# Supplementary material for: Wild salmon migration routes influence sea lice infestations: An agent-based model predicting farm-related infestations on juvenile salmon
Source: PLoS One. 2024 Aug 20;19(8):e0309215. doi: 10.1371/journal.pone.0309215 (PMC11335166; doi:10.1371/journal.pone.0309215)
Supplement: Attachment — Submitted filename: Review of manuscript by Jeong et al.pdf [file pone.0309215.s001.pdf]

**Review of manuscript by Jeong et al. for PlosOne**

The study presents a virtual post-smolt model simulating the migration and *Caligus clemensi* infestation of juvenile wild sockeye salmon in an archipelago in British Columbia, Canada. Three migration routes are compared. A finding highlighted in the abstract is that a longer migration route farther from farms led to higher lice loads on the juvenile salmon than a shorter route closer to the farms, underscoring that exposure time may be more important than infestation pressure in some cases. As the study is a modelling study, it depends critically on the assumptions in the model. The sensitivity analysis and discussion partly but not fully addresses these limitations.

Main comments:

The sensitivity analysis focuses on the correlation between different model setup parameters and the model output (the number of lice on salmon smolts). That is useful information but somehow difficult to interpret, since the correlation can depend strongly on how large parameter range is explored. Was that range standardised somehow, to make the correlations for different parameters comparable?

In addition, the correlations do not necessarily show the sensitivity of the conclusions drawn. In particular, I urge to also show results on how conclusions about differences between routes depend on modelling choices.

The sensitivity of the results to the logarithmic relationship between infestation pressure and infestation rate is discussed thoroughly, but as no sensitivity analysis on this model choice is presented, it remains unknown which results are indeed sensitive to this assumption. I strongly suggest to add a sensitivity analysis for this key model assumption. The data for this exist, as reference [31], in addition to the parameters for eq. 3, give parameters for an alternative model formulation that assumes a proportional relationship. The discussion relating to this relationship and the potential influence on results is otherwise very good.

Route 1 differs fundamentally from the other two routes in that fish are assumed to take the shortest route without meandering. Is it feasible to add a sensitivity analysis to this assumption, e.g. by assuming a faster swimming speed so that the fish had to meander in all routes? This comment can be ignored if the authors think that such an approach is unlikely to provide new insights.

The conclusions about differences in lice infestation along the three routes depend strongly on the progression rates estimated in reference [19]. Please provide a bit more information about sample size and reliability of these estimates in the Material and Methods, and some sentences about this uncertainty in the Discussion.

The presentation of the study is not clear enough. I have added a number of detailed comments and suggestions to explain what I found unclear.

Line 296: Please explain why you used a dispersion parameter (2.733) from a different model than where the parameters in eq. 3 came from. Consider adding a sensitivity analysis to this modelling choice.

Detailed comments:

Line 28: Unclear what “lower-level” means. Is it the same as “low”?

Line 92: I am not familiar with this species of sea lice. Can the wild salmon also be infested by other louse stages than the larvae? This question is not important for the robustness or validity of the modelling.

Section 1.2: I missed a description of the time scale of the model (1 out-migration season; time-step 4.63 min.).

Lines 122-125: This list seems a mix of state variables and higher-level model settings, but I am not familiar with how the ODD framework uses the term state variables.

Line 130: Each pixel is 100x100 = 10000 m^2, not 100 m^2.

Line 130: Is it correct with plural infestation pressure “values” here? That is confusing.

Line 131: *C. clemensi*

Line 174: I found this sentence confusing, as I read it as referring to an individual fish, and it was not understandable “consistent” between what. Perhaps rewrite to “... taken by different fish may differ ...” or similar.

Line 197: Is “transmission pressure” the same as other places referred to as “infestation pressure”?

Line 223: Each pixel is 100x100 = 10000 m^2, not 100 m^2

Lines 239-241: This sentence is confusing, as it seems to refer to the migration model in the submitted manuscript. Please make it clear from the beginning of the sentence that this refers to a completely different migration model. It is not at all clear to me why the migration model by Moriarty et al. is discussed here.

Lines 260-261: The rest of the paragraph would be easier to follow if you here wrote more explicitly that acoustically tagged juvenile sockey salmon have been found to have progression rates of X, Y and Z km/day in routes 1, 2, and 3, respectively [19], and that you used these values as basis for the simulations.

Line 286: “after”, or did the salmon reach the lice before they reached the farms?

Line 297 and elsewhere: I find it confusing that “count” is used as synonym for concentration or number. To me, a count refers to a number obtained by counting something – for example the number of lice counted on a physical sample of fish, which is generally not the case here.

Lines 328-354 (Sensitivity analysis): It was hard to understand what was done until after reading the results. It may help if you separate better what was sensitivity to model assumptions (e.g. sea lice abundance in farms), which implied rerunning the model multiple times, and what was postprocessing of model results to look for patterns in the variation between simulated salmon smolts in the baseline run. I am still not sure I have understood exactly how this was done.

Lines 339-341: I don’t understand how progression rates and meandering were modelled under the alternative assumptions about swimming speed. Or is this post-processing of results from the baseline model, as described for sensitivity to progression rates?

Line 387: How was the total infestation pressure calculated?

Lines 489-500: This paragraph does not seem to distinguish well between the total infestation, which is addressed by the sensitivity analysis, and infestation rate, which is addressed in the experiments in reference [35]. Please rewrite for clarity. Also, please write out the author (Samsing et al.) in the first sentence around line 489, so that it is immediately clear that the results discussed around line 499 is the same study and not another. Lines 501-502 seems to repeat previous points and lines 502-505 seem to be the mechanism behind [35]’s findings about infestation rate, and not “In addition”.

Lines 523-525: It is a misunderstanding that reference [15] required choosing between calibrating to trawl data and 10 km/day OR sentinel cage data and 7 km/day. Both trawl and cage data were used for both speeds, and the choice of which data to calibrate to can be made by setting the indicator variable for cage vs trawl to 0 or 1.
