# Supplementary material for: Wild salmon migration routes influence sea lice infestations: An agent-based model predicting farm-related infestations on juvenile salmon
Source: PLoS One. 2024 Aug 20;19(8):e0309215. doi: 10.1371/journal.pone.0309215 (PMC11335166; doi:10.1371/journal.pone.0309215)

PLOS ONE

Wild salmon migration routes influence sea lice infestations: An agent-based model predicting farm-related infestations on juvenile salmon

--Manuscript Draft--

Manuscript Number:	PONE-D-24-06375
Article Type:	Research Article
Full Title:	Wild salmon migration routes influence sea lice infestations: An agent-based model predicting farm-related infestations on juvenile salmon
Short Title:	Agent-based model for lice infestation
Corresponding Author:	Jaewoon Jeong, Ph.D.<br>Fisheries and Oceans Canada<br>Ottawa, ON CANADA
Keywords:	Aquaculture, Agent-based model, Infestation pressure, Salmon conservation, Sea lice, Sockeye salmon
Abstract:	This study presents an Agent-Based Model (ABM) simulation to assess the impact of varying migration routes on sea lice (Caligus clemensi) infestation levels in juvenile wild sockeye salmon (Oncorhynchus nerka) in the Discovery Islands, British Columbia, Canada. This research highlights the importance of migratory routes in determining the extent of exposure to sea lice originating from nearby salmon farms. Three northward out-migration routes were modelled, each exposing the fish to different levels of infestation pressure based on proximity to salmon farms. The ABM incorporates spatially explicit migration patterns of juvenile sockeye salmon using a detailed raster map of the Discovery Islands. Key variables such as swimming speed, progression rate, and infestation levels were integrated into the model, offering a comprehensive analysis of migration and infestation dynamics. The study revealed that infestation rate is highly variable, depending on migration routes. Specifically, salmon traveling longer migration routes farther from farms may experience higher sea lice loads than those on shorter, farm-adjacent routes. This underscores the role of lower-level infestation pressures and the critical influence of swimming speed, which affects exposure time, and thus infestation rates. Additionally, the study conducted a sensitivity analysis to understand the influence of various parameters on infestation rates. This analysis highlighted the importance of swimming speed and progression rate, particularly in routes closer to the farms. The findings suggest that slower swimming speeds and meandering routes increase exposure to lice, thereby elevating infestation levels. The research contributes to understanding the dynamics of sea lice transmission and its relationship with salmon migration patterns. It underscores the necessity of considering migratory routes and farm proximity in managing and mitigating the impact of sea lice infestation on wild salmon populations. This study's insights are crucial for developing strategies to balance aquaculture practices with the conservation of wild salmon.
Order of Authors:	Jaewoon Jeong, Ph.D.
	Gregor McEwan
Additional Information:	
Question	Response
**Financial Disclosure**<br><br>Enter a financial disclosure statement that describes the sources of funding for the work included in this submission. Review the submission guidelines for detailed requirements. View published research articles from *PLOS ONE* for specific examples.	Yes

This statement is required for submission and **will appear in the published article** if the submission is accepted. Please make sure it is accurate.<br><br>**Funded studies**<br>Enter a statement with the following details:<br>• Initials of the authors who received each award<br>• Grant numbers awarded to each author<br>• The full name of each funder<br>• URL of each funder website<br>• Did the sponsors or funders play any role in the study design, data collection and analysis, decision to publish, or preparation of the manuscript?<br><br>Did you receive funding for this work?	
Please add funding details.<br>as follow-up to "**Financial Disclosure**<br><br>Enter a financial disclosure statement that describes the sources of funding for the work included in this submission. Review the submission guidelines for detailed requirements. View published research articles from *PLOS ONE* for specific examples.<br><br>This statement is required for submission and **will appear in the published article** if the submission is accepted. Please make sure it is accurate.<br><br>**Funded studies**<br>Enter a statement with the following details:<br>• Initials of the authors who received each award<br>• Grant numbers awarded to each author<br>• The full name of each funder<br>• URL of each funder website<br>• Did the sponsors or funders play any role in the study design, data collection and analysis, decision to publish, or preparation of the manuscript?	Aquaculture, Biotechnology and Aquatic Animal Health Science Branch at the Fisheries and Oceans Canada provided a research supporting fund for this study.

Did you receive funding for this work?"	
Please select the country of your main research funder (please select carefully as in some cases this is used in fee calculation).<br>as follow-up to "**Financial Disclosure**<br><br>Enter a financial disclosure statement that describes the sources of funding for the work included in this submission. Review the submission guidelines for detailed requirements. View published research articles from *PLOS ONE* for specific examples.<br><br>This statement is required for submission and **will appear in the published article** if the submission is accepted. Please make sure it is accurate.<br><br>**Funded studies**<br>Enter a statement with the following details:<br>• Initials of the authors who received each award<br>• Grant numbers awarded to each author<br>• The full name of each funder<br>• URL of each funder website<br>• Did the sponsors or funders play any role in the study design, data collection and analysis, decision to publish, or preparation of the manuscript?<br><br>Did you receive funding for this work?"	CANADA - CA
**Competing Interests**<br><br>Use the instructions below to enter a competing interest statement for this submission. On behalf of all authors, disclose any competing interests that could be perceived to bias this work—acknowledging all financial support and any other relevant financial or non-financial competing interests.<br><br>This statement is required for submission	The authors have declared that no competing interests exist.

and **will appear in the published article** if the submission is accepted. Please make sure it is accurate and that any funding sources listed in your Funding Information later in the submission form are also declared in your Financial Disclosure statement.<br><br>View published research articles from *PLOS ONE* for specific examples.<br><br>**NO authors have competing interests**<br><br>Enter: *The authors have declared that no competing interests exist.*<br><br>**Authors with competing interests**<br><br>Enter competing interest details beginning with this statement:<br><br>*I have read the journal's policy and the authors of this manuscript have the following competing interests: [insert competing interests here]*<br><br>* typeset	
**Ethics Statement**<br><br>Enter an ethics statement for this submission. This statement is required if the study involved:<br><br>• Human participants<br>• Human specimens or tissue<br>• Vertebrate animals or cephalopods<br>• Vertebrate embryos or tissues<br>• Field research<br><br>Write "N/A" if the submission does not require an ethics statement.<br><br>General guidance is provided below. Consult the submission guidelines for detailed instructions. **Make sure that all information entered here is included in the Methods section of the manuscript.**	N/A

**Format for specific study types**<br><br>**Human Subject Research (involving human participants and/or tissue)**<br>• Give the name of the institutional review board or ethics committee that approved the study<br>• Include the approval number and/or a statement indicating approval of this research<br>• Indicate the form of consent obtained (written/oral) or the reason that consent was not obtained (e.g. the data were analyzed anonymously)<br><br>**Animal Research (involving vertebrate animals, embryos or tissues)**<br>• Provide the name of the Institutional Animal Care and Use Committee (IACUC) or other relevant ethics board that reviewed the study protocol, and indicate whether they approved this research or granted a formal waiver of ethical approval<br>• Include an approval number if one was obtained<br>• If the study involved *non-human primates*, add *additional details* about animal welfare and steps taken to ameliorate suffering<br>• If anesthesia, euthanasia, or any kind of animal sacrifice is part of the study, include briefly which substances and/or methods were applied<br><br>**Field Research**<br><br>Include the following details if this study involves the collection of plant, animal, or other materials from a natural setting:<br>• Field permit number<br>• Name of the institution or relevant body that granted permission	
**Data Availability**<br><br>Authors are required to make all data underlying the findings described fully available, without restriction, and from the time of publication. PLOS allows rare exceptions to address legal and ethical concerns. See the PLOS Data Policy and FAQ for detailed information.	Yes - all data are fully available without restriction

A Data Availability Statement describing where the data can be found is required at submission. Your answers to this question constitute the Data Availability Statement and **will be published in the article**, if accepted.<br><br>**Important:** Stating 'data available on request from the author' is not sufficient. If your data are only available upon request, select 'No' for the first question and explain your exceptional situation in the text box.<br><br>Do the authors confirm that all data underlying the findings described in their manuscript are fully available without restriction?	
**Describe where the data may be found in full sentences. If you are copying our sample text, replace any instances of XXX with the appropriate details.**<br><br>• If the data are **held or will be held in a public repository**, include URLs, accession numbers or DOIs. If this information will only be available after acceptance, indicate this by ticking the box below. For example: *All XXX files are available from the XXX database (accession number(s) XXX, XXX.)*.<br>• If the data are all contained **within the manuscript and/or Supporting Information files**, enter the following: *All relevant data are within the manuscript and its Supporting Information files.*<br>• If neither of these applies but you are able to provide **details of access elsewhere**, with or without limitations, please do so. For example:<br><br>*Data cannot be shared publicly because of [XXX]. Data are available from the XXX Institutional Data Access / Ethics Committee (contact via XXX) for researchers who meet the criteria for access to confidential data.*<br><br>*The data underlying the results presented in the study are available from (include the name of the third party*	https://github.com/jaewoonjeong/ABMsalmon

and contact information or URL).<br>• This text is appropriate if the data are owned by a third party and authors do not have permission to share the data.<br><br>* typeset	
Additional data availability information:	

Manuscript Click here to access/download;Manuscript;Main document.docx

Wild salmon migration routes influence sea lice infestations: An agent-based model predicting farm-related infestations on juvenile salmon

Jaewoon Jeong [1 *] and Gregor McEwan [2]

[1] Aquaculture, Biotechnology and Aquatic Animal Health Science Branch, Fisheries and Oceans Canada, 200 Kent Street, Ottawa, ON K1A 0E6, Canada

[2] Modail Mara Inc., Charlottetown, Prince Edward Island, Canada

*Corresponding author

E-mail: Jaewoon.Jeong@dfo-mpo.gc.ca (JJ)

Short title: Agent-based model for lice infestation

Abstract

This study presents an Agent-Based Model (ABM) simulation to assess the impact of varying migration routes on sea lice (*Caligus clemensi*) infestation levels in juvenile wild sockeye salmon (*Oncorhynchus nerka*) in the Discovery Islands, British Columbia, Canada. This research highlights the importance of migratory routes in determining the extent of exposure to sea lice originating from nearby salmon farms. Three northward out-migration routes were modelled, each exposing the fish to different levels of infestation pressure based on proximity to salmon farms. The ABM incorporates spatially explicit migration patterns of juvenile sockeye salmon using a detailed raster map of the Discovery Islands. Key variables such as swimming speed, progression rate, and infestation levels were integrated into the model, offering a comprehensive analysis of migration and infestation dynamics. The study revealed that infestation rate is highly variable, depending on migration routes. Specifically, salmon traveling longer migration routes

farther from farms may experience higher sea lice loads than those on shorter, farm-adjacent routes. This underscores the role of lower-level infestation pressures and the critical influence of swimming speed, which affects exposure time, and thus infestation rates. Additionally, the study conducted a sensitivity analysis to understand the influence of various parameters on infestation rates. This analysis highlighted the importance of swimming speed and progression rate, particularly in routes closer to the farms. The findings suggest that slower swimming speeds and meandering routes increase exposure to lice, thereby elevating infestation levels. The research contributes to understanding the dynamics of sea lice transmission and its relationship with salmon migration patterns. It underscores the necessity of considering migratory routes and farm proximity in managing and mitigating the impact of sea lice infestation on wild salmon populations. This study's insights are crucial for developing strategies to balance aquaculture practices with the conservation of wild salmon.

KEY WORDS: Aquaculture, Agent-based model, Infestation pressure, Salmon conservation, Sea lice, Sockeye salmon

Introduction

Maintaining aquaculture sources like salmon farming is crucial to meet the growing seafood demand [1]. However, salmon aquaculture has been linked to the declining populations of wild salmon [2]. A major concern is the transmission of sea lice from salmon farms to migrating juvenile wild salmon; juveniles are more vulnerable than adults due to their underdeveloped scales and immune systems, which offer less protection against parasites [3]. Such infestations in juvenile salmon may lead to a diminished number of adult salmon returning to their spawning grounds. Consequently, it becomes imperative to thoroughly evaluate the risk that salmon farms pose to wild salmon populations and implement measures to mitigate this risk while meeting the rising demand for salmon as a food source.

It is challenging to predict sea louse infestation in juvenile salmon during their migration through areas with sea lice presence. This complexity arises from an interplay of factors such as sea lice population dynamics, the speed and distance of salmon migration, and the effectiveness

of lice attachment to fish [4]. The complex behaviour of juvenile salmon during migration further complicates experimental trials. Nevertheless, computer models, especially spatially explicit Agent-Based Models (ABMs), offer a viable solution for simulating intricate spatial scenarios, such as salmon migration [4]. ABMs are valuable for incorporating the spatial aspects of the system and for simulating the stochastic movement of wildlife [5]. They are considered a promising tool for representing the stochastic nature of fish movement [6] and have been used to simulate the stochastic characteristics of wild juvenile salmon migration [7]. For instance, ABM can incorporate individual variability in progression rate and route choice [8]. An ABM was used to explore how environmental and intrinsic factors can modulate the effect of salmon lice on survival, growth and maturation of migrating Atlantic salmon smolts [9], as well as on the population dynamics of sea trout [10].

Infestation pressure is a crucial indicator of the lice infestation threat from farms to wild juvenile salmon. It is calculated by estimating the number of infective lice larvae in the water column, often employing data from lice counts on farmed salmon and models that simulate larval dispersion, based on environmental elements such as currents and tides [11]. However, the accurate quantification of sea lice infestation pressure presents challenges due to the complex life cycle of sea lice, their uneven spatial distribution of larvae and environmental variability [12]. Despite these challenges, the integration of direct measurement with modelling has been enhancing the precision of these estimates [13]. Models estimate sea lice infestation rates at a particular location by calculating the infestation pressure, considering the number of sea lice at neighbouring farms and their proximity to these farms [9, 14, 15]. In determining the relationship between infestation pressure and the infestation rate on juvenile salmon, lice counts from sentinel cages or trawls were used to align with the assessed infestation pressure [16].

In this study we developed an ABM to simulate the out-migration of juvenile sockeye salmon in the Discovery Islands, British Columbia (BC), Canada. Sockeye salmon (*Oncorhynchus nerka*), after one or two years in freshwater, mature into smolts adapted for saltwater life and migrate downstream towards the ocean [17]. This species is the dominant Pacific salmon passing through the Discovery Islands region in BC [18]. Recent research utilized acoustic tagging to gain deeper insights into these migration patterns [19]. Their findings indicate significant variability in the distance travelled and the speed of migration, depending largely on the chosen

routes. The unique geographical features of the Discovery Islands, with their channels and peninsulas, offer a variety of migration seaways. The choice of these routes can lead to varying levels of exposure to nearby salmon farms, suggesting that the fine-scale migratory routes are crucial in determining the rate of lice infestation [20].

In this study, we present results from an Agent-Based Model simulating juvenile sockeye salmon migration through multiple fine-scale routes in the Discovery Islands, BC, Canada. These routes expose the salmon to larval lice originating from nearby salmon farms. Our model evaluates how different migration routes affect sea lice infestation rates on out-migrating wild juvenile salmon. Additionally, we conduct sensitivity analysis to identify key factors influencing the infestation rate. The analysis helps us understand the uncertainty and potential implications of various parameters on the rate of infestation, thereby offering a deeper insight into the dynamics of sea lice transmission in this context.

Materials and Methods

The model, developed in R [21], is an agent-based simulation that captures the out-migration of juvenile wild salmon in an offshore region, including potential infestations by larval lice during their migration. The source code is available at https://github.com/jaewoonjeong/ABMsalmon.

To describe our model, we have used the ODD (Overview, Design concepts, and Details) protocol [22]. In Overview, we introduce the model's agents. In Design concepts, we describe the general principles of the model's design. Details explains how fish migrate in the region and how they are infested with lice, including the equations that govern how the model operates.

1. Overview

1.1. Purpose

The primary objective of our model is to examine the impact of different out-migration routes on sea lice infestation levels in juvenile wild salmon. Focusing on a case study, we consider the scenario in which juvenile sockeye salmon encounter *Caligus clemensi* while migrating through the Discovery Islands, BC, Canada. We specifically consider *C. clemensi* as the primary

infesting species over *Lepeophtheirus salmonis*, as it is the dominant lice species found on sockeye salmon in this region [18, 23]. The model highlights how varying migration routes lead to differing degrees of exposure to sea lice from nearby salmon farms. Thus, we simulate the salmon navigating through these routes to investigate the consequent impact on infestation levels by the end of their migration. Additionally, through a sensitivity analysis, we explore the influence of various migration-related parameters and proximity to salmon farms on the abundance of lice in wild salmon.

1.2. Entities, state variables and scales

The entities of the models are individual juvenile sockeye salmon. The state variables describing the salmon include fish location (longitude and latitude), swimming speed (km/day), progression rate (km/day), migration duration (days), calendar date since the start (days since June 1st), migration distance (km), and infestation level (number of lice per fish).

Our model provides a spatially explicit depiction of the offshore migration routes of juvenile sockeye salmon in the Discovery Islands (**Fig 1**). To achieve this, we created a detailed raster map of the Discovery Islands, with each pixel measuring 100m x 100m, enabling comprehensive quantitative mapping along the coastline [14]. This mapping utilized the R package ‘raster’ [24]. Each 100 m^2 pixel is characterised by infestation pressure values, which indicate the concentration of *C. Clemensi* copepodid larvae present. The mechanism by which salmon entities increase their infestation level upon encountering these larvae is detailed below.

Fig. 1. **The study region on the Discovery Islands, British Columbia, Canada.** The map displays eight salmon farms (F1-F8) represented as black squares. Juvenile sockeye salmon are simulated to migrate from the Start Point to the End Point. Infestation pressure refers to the local concentration of *Caligus clemensi* copepodid larvae per 100m x 100m grid cell.

Typically, sockeye salmon from the Fraser River embark on their journey into the Strait of Georgia during spring [23]. Once in the ocean, their migration pattern generally leads them northward towards the North Pacific Ocean. They usually reach the Discovery Islands area around May and June [25]. We based our design of the three northward out-migration routes on the findings of research that tracked acoustically tagged juvenile sockeye salmon as they

navigated through the Discovery Islands [19]. In our simulations, all individuals are assumed to be released on the same day, the 1st of June. This assumption simplifies the out-migration of juvenile sockeye salmon in Discovery Islands originating from Fraser river. However, since the infestation pressure in our model does not include temporal dynamics, this simplification has minimal impact on the final outcome.

1.3. Process overview and scheduling

We established three out-migration routes, each allocated 3000 fish, between the start and end points. To estimate the salmon's swimming speed, we assumed that fish traveling through Discovery Passage follow the most direct seaway distance without meandering. Based on this assumption, we calculated a baseline swimming speed of 31.1 km/day. This calculation is derived from the average speed of sockeye salmon traversing the Discovery Passage in 2017 and 2018 [19]. This speed is consistent with the previously observed range of 0.2 – 0.5 m/s [26].

Given our model's specific focus on examining the impact of different migration routes, we intentionally omitted certain biological characteristics of juvenile wild fish, like body size and body growth. This deliberate exclusion is based on the rationale that such factors would uniformly affect fish across all migration routes, and our primary interest lies in isolating and analysing route-specific impacts.

2. Design concepts

This section outlines the fundamental principles underlying the model's design. Within the framework of the ODD protocol we employ to describe our model, various topics are defined in the design concepts section, including Emergence, Adaptation, Objectives, Sensing and Stochasticity.

2.1. Emergence

We are interested in one emergent property of the model: the infestation of sea louse on juvenile wild salmon during their out-migration.

2.2. Adaptation

Individual salmon are allowed to move to one of four pixels of north, south, west or east.

2.3. Objectives

Individuals are simulated to migrate towards the endpoint using one of three possible out-migration routes. The specific route taken by individual fish may not be consistent due to stochastically modelled migration.

2.4. Sensing

Individual fish are assumed to be able to sense the seaway distance from those four possible next locations to the end point. They use this distances to make the probability-weighted sampling, which determines their next location of pixel.

2.5. Interaction

Our model only investigates the impact of different migration routes, so the salmon do not interact with each other. Any influence that other salmon may have on each other's paths are simplified to be included in our 'meandering' variable, which gives us explicit control.

2.6. Stochasticity

Empirical observational studies using tagging approaches have shown great variability in migration speed. Therefore, we added a stochastic element to the model to create individual variability in migration speed and fine-scale migration seaways [27], which we refer to as 'meandering'. The mechanism works as follows: based on the distance from four points to the endpoint, the salmon are most likely to choose the location with the shortest distance to the endpoint. The location with the second shortest distance has the second highest probability, and so on. We varied the distribution of probabilities for these four potential next locations on each route to align the model's migration distance and period with the actual observational data of sockeye salmon in the Discovery Islands [19]. An advantage of making meandering an adjustable input variable is that it enables us to explore the effects of varying spatial aspects of salmon swimming behaviours on infestation levels.

Moreover, the sea lice infestation in our model is treated stochastically, being determined by a negative binomial distribution. The transmission pressure in each pixel serves as the mean parameter for this distribution, a process which is elaborated in the 'Details' section.

2.7. Observations

The primary output from the model is the count of sea lice infestations on individual salmon. In addition, the model records the period and distance of their migration.

3. Details

3.1. Initialization

In each simulation run, an individual fish begins its migration at the starting point and swims at a speed of 31.1 km/day. The fish is deterministically assigned one of the three routes.

3.2. Input data

The model's input includes infestation pressure, imposed by all eight salmon farms, applied to each pixel within the study region.

3.3. Sub-models

3.3.1. Migration model

During May and June of 2017 and 2018, eight active Atlantic salmon farms were identified in the study region [28], coinciding with the period when tracked the migration of juvenile sockeye salmon in the Discovery Islands [19] (**Fig 1B**). Of these, four farms were clustered in the Okisollo Channels, while three were more dispersed in the Calm-Cordero Channel. Notably, no farms were present in Discovery Passage. One farm is positioned near the endpoint. As a result, each of the three migration routes exposed the salmon to varying degrees of proximity to these farms. Route 1 offered the least exposure, being relatively distant from the farms. In contrast, Route 2 passed very close to four farms, and Route 3 mostly avoided immediate proximity to the farms.

In the model, we assumed that traversing each pixel represents a uniform 100-meter journey, either in a north-south or east-west direction, explicitly excluding diagonal paths (**Fig 2**). This design is based on the 100 square meter size of each pixel [14]. To navigate, individual fish use a raster map formatted into a 100m x 100m grid. In the open ocean segment of the model, each pixel is assigned a seaway distance value to the endpoint [27], which the fish use to

stochastically determine their migration route. At each movement step, the fish assess distances to the endpoint from their four adjacent cells to select their subsequent direction.

Fig. 2. **Two examples of stochastic migration paths of juvenile sockeye salmon.** These paths demonstrate their journeys along Route 3, near farm F1 (as detailed in Fig. 1). The sample salmon traveled from east to west, with closed and open circles indicating the pixels they traversed. Each pixel in the grid measures 100m x 100m. The salmon spent approximately 4.63 minutes in each cell, maintaining an equal duration across all cells.

If fish were to take the shortest seaway path from start to end point, the migration distances for Routes 1, 2, and 3 would be 106.7 km, 113.7 km, and 143.7 km, respectively, corresponding to migration durations of 3.4 days, 3.7 days, and 4.6 days. However, realistically, fish are highly unlikely to follow such direct routes and may meander, incorporating an element of randomness in their movement. To account for this in the salmon migration model, mathematical equation was implemented that integrates random movements with a specific directional term, thus guiding the fish to move in their intended direction [20]. In contrast, we utilized a raster map to simulate the movement of individual fish, aiming for a more spatially explicit approach.

To account for this variability in movement, we integrated concepts of swimming speed and progression rate into our model. The swimming speed was defined as the distance travelled between two points divided by the migration period [8], expressed as:

$$Swimming\ speed = \frac{D_{lm}}{t} \quad (1)$$

The progression rate was calculated as the shortest seaway distance between two points divided by the migration period, represented as:

$$Progression\ rate = \frac{d_{lm}}{t} \quad (2)$$

Here, D_{lm} denotes the actual movement distance of fish between two points of k and l, d_{lm} signifies the shortest seaway distance between these two points, and the migration period, t, is consistently applied to both calculations.

When a fish takes the most direct seaway route between two points, its swimming speed matches the progression rate. However, if the fish opts for a route that deviates from the most direct seaway path, the distance covered, as indicated by its swimming speed, will be greater than the progression rate. As such, swimming speed can be considered a theoretical and perhaps less pragmatic measure when compared to the progression rate. A significant disparity between the swimming speed and progression rate indicates prolonged meandering by the fish during its migration, resulting in covering extra distance. Given that the swimming speed remains consistent throughout each simulation, the migration distance is directly proportional to the migration period in our baseline model.

Progression rates differ across migration routes. Route 1 exhibits the highest rate, while Route 3 shows the lowest [19]. Since only progression rates were recorded for acoustically-tagged juvenile sockeye salmon, we assumed a uniform swimming speed of 31.1 km/day, in line with the observed progression rate for Route 1 [19]. In our model, we designate one time step as 4.63 minutes, during which a fish traverses 0.1 km. The swimming speed is consistent across all routes throughout the simulation. We introduced variation in progression rates using probability-weighted sampling, influenced by the meandering parameter, α. As fish decide which next pixel to move to among four possible directions (north, south, west, and east), the choice probability is dictated by the distance to the endpoint, with the pixel at the shortest distance being the most likely selection. For the upcoming four pixels in the subsequent time step, the probability for each is defined in the sequence of $1-\alpha$, $\alpha/2$, $\alpha/2$, and 0, based on their increasing distances to the endpoint. The α values were set at 0, 0.29, and 0.40 for Routes 1, 2, and 3, respectively, calibrated to match the model's average progression rates with those observed [19].

3.3.2. Infestation model

The infestation model works with the migration model to determine the likelihood of infestation of the migrating wild juvenile sockeye salmon. In a simplified approach, the infestation likelihood is determined by multiplying the attachment rate, which indicates the frequency of successful lice infestation post-contact, with the encounter rate, representing the frequency of contact between fish and lice [29]. The attachment rate is influenced by sea lice biology, host fish attributes, and environmental conditions [30]. In contrast, the encounter rate hinges on the

sea lice concentration. To emphasize the impact of salmon farms, we excluded variables associated with the attachment rate, assuming every encounter results in infestation.

It was demonstrated that markedly higher *C. clemensi* abundance among salmon migrating through salmon farms in the Discovery Islands compared to those migrating through a salmon farm-free area on the north coast of British Columbia [23]. Additionally, they noted a pronounced increase in *C. clemensi* abundance in salmon sampled before passing through the farms in the Discovery Islands, suggesting a potential role of open net-pen salmon farms in transmitting *C. clemensi* to wild juvenile sockeye salmon. Therefore, in our model, we assumed that all lice infestations originated from *C. clemensi* copepodids associated with salmon farms to specifically examine the impact of salmon farms on lice infestation in juvenile sockeye salmon.

The output of our ABM is the sea lice count on fish at the migration endpoint. This count is derived as the cumulative sum of random numbers assigned at each pixel that the fish traversed. These numbers are stochastically selected from a negative binomial distribution, which is characterized by its mean (μ) and a dispersion parameter (κ). Consequently, the total sea lice count results from the summation of these numbers throughout the migration process. We used μ_i to represent the number of lice encounters at pixel i, and κ was set at 2.733 based on observed salmon lice infestation data [14]. Infestation pressure represents the count of infective larvae at a specific location [15]. μ_i was derived from the function of infestation pressure, IP_i, at pixel i, in relation to swimming speed. To estimate infestation pressure, specifically the copepodid count at a distinct pixel, we used the total copepodid count from all eight farms along with a kernel density function [31]. The calculation of μ_i followed this equation [15].

$$\mu_i = \exp\left(-5.49 + log\left(\frac{0.1}{Speed}\right) + 0.425 * log(IP_i)\right) \tag{3}$$

Here, 'Speed' represents the swimming speed of juvenile sockeye salmon at 31.1 km/day. The term $log(0.1/Speed)$ adjusts the equation to account for the duration a migrating fish spends at a pixel. IP_i, which represents the infestation pressure at pixel i, offers an estimate of the copepodids present with a fish for a specific duration at that pixel. The cumulative infestation pressure across all traversed pixels provides a comprehensive measure of the infestation risk to the fish during its out-migration, originating from copepodids associated with the eight salmon

farms in the region. Infestation pressure IP_i at pixel i, resulting from all eight farms in the study region, was calculated using the following equation,

$$IP_i = \sum_{j=1}^{8} X_{i,j} \tag{4}$$

The pressure exerted from farm *j* at pixel *i* ($X_{i,j}$) was derived from,

$$X_{i,j} = \frac{W(d_{i,j})}{\sum_{i=1}^{n} W(d_{i,j})} * N \tag{5}$$

Here, "n" denotes the total pixel count. "N" refers to the copepodid lice count per farm. The copepodid tally from the farms gets allocated across pixels, contingent upon the seaway distance between each farm and all the study area locations, utilizing a kernel density function [4]. This equation employs the Gaussian kernel density to apportion copepodid numbers from farms based on the seaway distance from farm j to pixel *i*.

In our model, all farms maintain a uniform sea lice count, which remains constant throughout the simulations. We assumed that each farm releases an identical total of 62.5 million copepodids, summing to 500 million from the eight farms. This value was selected to ensure that our model replicates sea lice abundance levels comparable to those in [23].

The function $W(d_{i,j})$ was derived using the Gaussian kernel density function. This function reflects the diminishing weight as the seaway distance between locations increases.

$$W(d_{i,j}) = \frac{1}{\sqrt{2\pi}(\sigma/4)} e^{-\frac{{d_{i,j}}^2}{2(\sigma/4)^2}} \tag{6}$$

Here, W represents the Gaussian kernel density's estimated weight for the seaway distance $d_{i,j}$, which signifies the seaway distance between the pixel *i* and salmon farm *j*. We assumed a fixed bandwidth (σ) of 30 km, representing the maximum distance of larval lice dispersal from their origin point [32]. Seaway distances between each combination of pixels and farms were calculated using the 'gdistance' package [33] in the R statistical language.

3.3.3. **Sensitivity analysis**

We performed a sensitivity analysis on our model to assess the influence of key parameters on the modelling outcomes, specifically the counts of sea lice per fish. These parameters

encompassed various factors, such as the chosen route, sea lice count per farm, bandwidth, swimming speed, and progression rates. Notably, in the estimation of infestation pressure, the sea lice count from each farm and the bandwidth within the Gaussian kernel density function played critical roles.

We simulated variations in sea louse counts ranging from one-twelfth to twice the baseline copepodid count, covering a broad spectrum of likely copepodid fluctuations. A reasonable range of bandwidths ranging 5 km to 100 km were explored [32]. Our sensitivity analysis included an examination of fish migration swimming speeds and progression rates. This decision was based on observed variations in travel speeds among individual sockeye salmon during coastal migration in southern British Columbia, Canada [8, 34]. The swimming speeds in our simulations ranged from 6.2 km/day (0.2 times the baseline speed) to 46.7 km/day (1.5 times the baseline speed) [35]. For Routes 2 and 3, the progression rate range in our sensitivity analysis was determined by the minimum and maximum rates observed across the 3000 simulations of the baseline model. In contrast, the progression rate for Route 1 was set at a constant 31.1 km/day.

In our sensitivity analysis, simulations were conducted by randomly selecting values for each parameter within their pre-defined ranges. The analysis comprised 3000 simulations in total. To evaluate the sensitivity of the model's outcomes to different parameters, we utilized Pearson's correlation coefficient (*r*). This coefficient measures the linear relationship between a model parameter and its output, with values spanning from -1 to 1. A value of 1 indicates a direct proportional increase, −1 reflects an inverse relationship, and a value near 0 suggests a negligible or weak correlation. Subsequent to the initial sensitivity analysis, which covered all routes and four key parameters, we conducted an in-depth investigation into the sensitivity specific to each route. This was to gain a clearer understanding of how the characteristics of each route uniquely impact the overall model.

Table 1. **Mean migration distance, migration duration, swimming speed, progression rate and total infestation pressure for each route.** These values were derived from the averages of 3000 simulations per route.

Route	Migration distance (km)	Migration period (days)	Swimming speed (km/day)	Progression rate (km/day)	Total infestation pressure (in millions)
**1**	106.7	3.4	31.1	31.1	44.3
**2**	196.8	6.3	31.1	18.1	171.1
**3**	373.3	12.0	31.1	12.1	150.8

Results

Juvenile salmon out-migration

Fig 3 presents ABM simulation depicting out-migration examples along three routes for juvenile sockeye salmon in the Discovery Islands. Unlike Route 1, where all simulated fish deterministically took the shortest seaway of 106.7 km, the migration distances for Route 2 ranged from 153.4 to 282.7 km and for Route 3 ranged from 281.5 to 513.7 km (**Fig 4A**). The mean migration distances for Route 2 and 3 were 196.8 and 373.3, respectively (**Table 1**). Thus, fish through Route 2 and 3 in our model took on average 1.73 (196.8 km / 113.7 km) and 2.60 (373.3 km / 143.7 km) times more migration distance than the shortest seaway of each route, respectively. While the progression rate for Route 1 was 31.1 km/day in every simulation, the progression rates for Route 2 ranged from 12.5 to 23.1 km/day and for Route 3 ranged from 8.7 to 15.9 km/day (**Fig 4B**).

Fig. 3. Example of five juvenile sockeye salmon stochastic migration paths for each route in the Discovery Islands, British Columbia, Canada. The map marks eight salmon farms with black squares and delineates each migration route from the start (bottom-right black circle) to the end (upper-left black circle). In every simulation, Route 1 deterministically adheres to the shortest path, resulting in an identical route marked by straight lines and right angles across all simulations. Conversely, Routes 2 and 3 show meandering salmon migration patterns, attributable to the stochastic nature of their path selection. Infestation pressure is applied in a manner identical to that shown in Fig. 1.

Fig. 4. **Migration Metrics Distribution Across Simulations.** For 3000 simulations of an agent-based model, the distributions of (A) migration distance, (B) progression rate, and (C) total infestation pressure are presented. In all simulations, Route 1 showed consistent results, marked by vertical dashed red lines.

Total infestation pressure of Route 1 was 44 million, and total infestation pressure of Route 2 and 3 ranged from 136 million to 274 million and from 95 million to 224 million, respectively (**Fig 4C**). Although Route 2 has salmon farms in closer proximity than Route 3, its total infestation pressure was only marginally higher. This is attributed to Route 3's longer migration distance, resulting from both its inherently longer seaway and its slower progression rate. **Fig 5** illustrates the cumulative infestation pressure per pixel for out-migrating fish from start to end, based on five examples of the simulations. Fish on Route 1 faced a sharp increase in infestation pressure near the four farms in the Okisollo Channel (**Fig 1B**), but their exposure duration was the shortest and subsequently the cumulative infestation pressure was the smaller than the other two routes. Fish on Route 2 encountered the most substantial rise in infestation pressure, especially when they migrated through the four farms in the Okisollo Channel. Conversely, those on Route 3 had a notable increase in infestation pressure as they moved through the Calm-Cordero Channel, but this was not as a sharp increase as Route 2. Yet, fish on Route 3 had a longer exposure, covering a distance about twice that of Route 2. Notably, the variations between routes were much more noticeable than the individual variations within the same route.

Fig. 5. Pixel-wise infestation pressure during juvenile sockeye salmon migration. This figure illustrates the cumulative infestation pressures encountered in five representative simulations across three different migration routes. The five simulations in each route are signified by a distinct colour. Route 1 simulations display uniformity, reflecting deterministic migration patterns, while Routes 2 and 3 exhibit variations due to stochastic influences.

Sea lice abundance by migration routes

In general, fish on Route 1 exhibited markedly lower abundance, and fish on Route 2 yielded slightly lower sea lice abundance than fish on Route 3 (**Fig 6**). The primary factor contributing to sea lice infestation on fish in Route 2 was the heightened total infestation pressure due to its closer proximity to salmon farms. In contrast, the elements enhancing sea lice infestation on fish through Route 3 were its longer migration distance and a slower progression rate, which extended the duration of exposure to lice from farms. Consequently, even though Route 2 had a

greater total infestation pressure compared to Route 3, the significantly extended migration period of Route 3 led to it having a higher average lice abundance than Route 2.

Fig. 6. **Sea lice abundance variation across three migration routes.** Histograms represent the distribution of sea lice infestations for juvenile sockeye salmon across three different migration routes, based on 3000 simulations.

Within each route, stochasticity was a predominant factor influencing the sea lice count per fish. For Routes 2 and 3, the correlation coefficients linking the sea lice count per fish to total infestation pressure were 0.14 and 0.12, respectively. Similarly, when associating lice abundance with migration distance, the coefficients were 0.08 for Route 2 and 0.07 for Route 3. These modest positive values suggest that while total infestation pressure and migration distance had an influence on the sea lice count per fish, their roles were not paramount. Instead, stochastic encounters largely dictated infestation dynamics. The substantial variation in the sea lice counts per fish across all routes further underscores the inherent stochasticity of these infestation events (**Fig 6**).

Sensitivity analysis

The tornado graphs (**Fig 7**) display the strength and direction of the relationship of each parameter to the model output. In **Fig 7A**, bars representing sea lice count, bandwidth, and Routes 2 and 3 extend to the right, showing that as the parameter values increase, the model output does as well. Conversely, bars representing swimming speed and progression rate extending to the left suggest an inverse linear relationship. Except for progression rate, the four parameters demonstrated a comparable influence on the model output regarding sea lice abundance.

Fig. 7. **Sensitivity analysis of parameters affecting salmon infestation using Pearson correlation coefficients.** (A) Comparative impact assessment of various parameters and route selection; Route 2 and Route 3 denote the relative impact of selecting these routes as opposed to Route 1. (B-D) Detailed impacts of parameters on infestation rates for individual routes: (B)

depicts Route 1 where the progression rate is constant; thus, it is excluded from analysis, (C) and (D) show the variable influences on Routes 2 and 3, respectively. Bars oriented to the right signify a direct correlation between parameter value and model output. Conversely, bars to the left represent an inverse correlation. The length of the bar indicates the strength of the correlation, with parameters organized in descending order of influence.

In our sensitivity analysis for each route, the influence of individual parameters varied. In the analysis of Route 1 (**Fig 7B**), bandwidth stands out as the variable with the most significant impact on the model's output, distinguishing it from Routes 2 and 3. In Routes 2 and 3 (**Figs. 7C and D**), swimming speed is identified as the most impactful variable on the model, with progression rate being the least. Notably, in Route 2, sea lice count was more influential than bandwidth, whereas in Route 3, bandwidth was more influential than sea lice count. Another finding between Routes 2 and 3 is that swimming speed and progression rate had a more marked influence in Route 2 compared to Route 3. Conversely, the effects of sea lice count and bandwidth were somewhat subdued in Route 2 relative to Route 3, even though the disparity was modest.

Discussion

Building on the observations of juvenile sockeye salmon migration in the Discovery Islands, BC, Canada by [19], we examined sea lice infestations along different out-migration routes, quantifying their exposure to salmon farms. Our investigation focused on the impact of route-specific exposures on sea lice abundance in wild salmon. For this purpose, we utilized an agent-based model to simulate *C. clemensi* infestation during the out-migration of juvenile sockeye salmon in the region. The results from our simulations underscored that the sea lice abundance on wild salmon can vary markedly based on their specific migration routes, even within one region. This disparity in sea lice abundance, stemming from differing exposure levels to salmon farms, is mainly influenced not only by the count of sea lice originating from farms but also by the duration of exposure between the salmon and sea lice. Integrating insights from deterministic model of sea lice distribution and stochastic model of juvenile salmon out-migration enables to predict the potential risk of pathogen-host interactions and to estimate the probable lice loads on out-migrating juvenile salmon with a focus on spatial scale [20]. However, our findings indicate

that inherent stochastic factors in lice infestation markedly influence infestation rates, leading to marked variability in sea lice counts across individual fish.

In our research, we refined the modelling of salmon migration patterns using progression rates, which accounted for the meandering pathways they often take, as opposed to previous studies that presumed direct trajectories [9, 14]. This adjustment provided a more realistic depiction, given that during their coastal migration, juvenile salmon frequently exhibit progression rates much lower than their actual swimming speeds due to varied behavioural strategies [8]. Notably, these rates varied depending on the migration routes [19]. For instance, salmon on direct routes like Route 1 progressed faster than those on more winding paths such as Route 3. This distinction is significant, as it suggests that salmon farms situated along meandering routes might present a higher risk of lice infestation compared to those on more linear paths.

The results of the sensitivity analyses emphasize the importance of swimming speed in determining sea lice infestation rate. To investigate the effect of varying swimming speed on the infestation rate, experiments were conducted using cylindrical raceways, where fish swam against the current [35]. Their findings revealed that lice attachment was considerably affected by swimming speed, with infestation levels being 2.5 times higher in moderate speed (17.0 cm/s) compared to high speed (36.7 cm/s) and 1.3 times higher compared to low speed (5.1 cm/s). However, it should be noted that this experiment used an equal period for each speed, which would make different swimming distances for each speed group. If an equal swimming distance was applied for all three groups of speeds, mean sea lice per fish would have been 4.6, 1.9 and 0.3 for low, moderate and high swimming speed, respectively. This result aligns with our finding that slower swimming speeds lead to higher sea lice infestation rate. In Samsing *et al*., a higher current velocity level increased sea lice abundance due to an elevated encounter rate [35]. However, given the same swimming distance, encounter rates were higher at low current velocities compared to high velocities. In addition, the low current velocity facilitated the attachment of larval lice to host fish. This increased propensity for attachment at slower speeds might be another factor contributing to the rise in sea lice abundance, a factor not accounted for in our model.

Also, the results from sensitivity analysis discovered that swimming speed and progression rate had a greater influence on Route 2 than on Route 3. This indicates that parameters linked to the duration of salmon exposure to lice are more significant in shorter routes near farms. In contrast, the heightened impact of sea lice count and bandwidth on Route 3, compared to Route 2, implies that factors related to infestation pressure are more impactful on longer routes located further from farms. This finding is attributed to the logarithmic relationship between infestation pressure and rate; variations in the lower infestation pressure of Route 3 are more noticeable than those in the higher infestation pressure of Route 2 [15].

The encounter rate function, represented by Eq. 3, defines the relationship between the sea lice count near fish and the likelihood of their encounter. Its significance in modelling results has been underscored in prior studies [9, 14, 15]. To construct this function, data has typically been drawn from two methods: lice infestation on migrating juvenile wild salmon observed through trawling [27] and sentinel cages [14, 16]. Each method, however, presents its own set of advantages and limitations. Notably, infestation rates from trawling data were found to be nearly ten times higher than those from sentinel cages [15]. In our study, we adopted the model based on trawl-caught wild juvenile salmon as presented in Stige *et al.* [15]. We chose this model for two reasons. First, the *C. clemensi* per juvenile sockeye salmon data from Price *et al.* closely aligned with the trawling data over the sentinel cage data [23]. Second, the assumed migration speed for the trawl data (10 km/day) was more in line with our model's progression rates compared to the 7 km/day speed assumed for sentinel cage data. While our choice was suitable for our specific context, the critical nature of this relationship highlights the ongoing need to enhance the encounter rate function's accuracy. Improved precision in this function is essential for a deeper understanding of the dynamics of sea lice infestation.

As explained by the encounter-dilution effect [36], an individual fish’s risk of parasitism increases with the rising number of parasites, but the increase is not linear. The encounter rate function, as delineated by Eq. 3, shows a logarithmic escalation in lice per fish with increasing infestation pressure [15]. Thus, variations in lower infestation pressures have a more pronounced effect, while the impact lessens with changes in higher pressures. A possible reason for this phenomenon is that individual infective copepodids have specific attachment preferences on their host fish, leading to increased competition among sea lice for prime attachment sites [37].

Moreover, a surge in attached lice might intensify the host fish's immune response, thus curbing subsequent infestations [38]. Additionally, consider a hypothetical zone around a sea louse in which it can infest a fish. This zone might intersect with those of other lice, especially as lice density increases [39]. As a result, a rise in lice count leads to a logarithmic increase in the collective area of these zones, offering a theoretical basis for the logarithmic relationship between infestation pressure and infestation rate.

To effectively address sea lice infestation on out-migrating juvenile salmon due to salmon farms, strategies could focus on either reducing lice release from farms or shortening the duration of exposure of wild salmon to these lice. Approaches to reduce lice counts may include regulating the number and biomass of farms and controlling sea lice levels on the farms. Meanwhile, reducing exposure time may involve managing the spatial density of salmon farms. Given the logarithmic relationship between infestation pressure and rate, farms in close proximity may result in a lower infestation rate on wild fish. However, spacing salmon farms apart is advantageous for reducing the transmission of sea lice between farms [40]. Further research is necessary to understand the collective impact of farm density. Additionally, the preferences of out-migrating fish for certain routes, influenced by factors like surface currents and wind, should be taken into account [41]. sockeye salmon in the Discovery Islands, for example, show a preference for Route 2 over Route 3 [19]. These fine-scale migratory patterns could be crucial in efforts to minimize lice exposure from farms. Moreover, considering the geographical features when locating farms is significant; the features of juvenile salmon migration routes can affect their migration speed. Positioning farms along more direct coastlines, such as Route 1, where wild salmon tend to migrate more directly and potentially faster, could reduce the duration of lice exposure [19].

Our study examined the dynamics of *C. clemensi* infestation on sockeye salmon within the Discovery Islands, BC, Canada. Our study parameters were based on farm locations and wild salmon migration in 2017 and 2018. However, since 2020, the region has seen a systematic removal of open-net salmon farms due to a governmental prohibition on restocking [42]. Consequently, the aim of our research was not to evaluate the present-day risk of sea lice infestation on wild sockeye salmon in the Discovery Islands. Instead, our goal was to provide insights potentially transferable to similar ecological systems elsewhere. It is important to

recognize that the model outcomes of this study are heavily dependent on Eq. 3, which postulates a logarithmic relationship between infestation pressure and encounter rate. Furthermore, the parameter ranges assumed in the sensitivity analysis are crucial to the results obtained. Since model outputs can vary significantly with different parameters [15], the equations and parameter values related with the dynamics of sea lice infestation on wild salmon necessitate additional refinement to enhance the accuracy of assessing the risk posed by farm-origin sea lice to juvenile wild salmon.

In our research, we utilized an ABM to address the spatially stochastic patterns in salmon migration. Building on this, it would be advantageous to expand the application of ABM for modelling the spatial dispersal of larval lice [43]. This extension would involve integrating ABM with hydrodynamic modelling, moving beyond the sole use of a kernel density function for estimating the spatial distribution of larval lice counts. Such a combined approach would enable to more accurately model the spatiotemporal variations in larval lice concentrations, adapting to the specific local environmental conditions [20].

Acknowledgments

We are grateful to Dr. Derek Price for his thorough review of our manuscript and for offering invaluable feedback.

Data Availability Statement

The source code of the agent-based model can be downloaded from https://github.com/jaewoonjeong/ABMsalmon.

Author Contributions

Conceived and designed the experiments: JJ GM. Performed the experiments: JJ. Analysed the data: JJ. Wrote the paper: JJ GM. Contributed to the design of the model: JJ GM. Model Implementation: JJ.

References

1. Smith MD, Roheim CA, Crowder LB, Halpern BS, Turnipseed M, Anderson JL, et al. Sustainability and global seafood. Science. 2010;327(5967):784-6.
2. Dadswell M, Spares A, Reader J, McLean M, McDermott T, Samways K, et al. The decline and impending collapse of the Atlantic Salmon (Salmo salar) population in the North Atlantic Ocean: a review of possible causes. Rev Fish Sci Aquac. 2022;30(2):215-58.
3. Torrissen O, Jones S, Asche F, Guttormsen A, Skilbrei OT, Nilsen F, et al. Salmon lice–impact on wild salmonids and salmon aquaculture. J Fish Dis. 2013;36(3):171-94.
4. Petersen JH, DeAngelis DL. Dynamics of prey moving through a predator field: a model of migrating juvenile salmon. Mathematical Biosciences. 2000;165(2):97-114.
5. Tang W, Bennett DA. Agent- based modeling of animal movement: a review. Geography Compass. 2010;4(7):682-700.
6. Mawer R, Pauwels IS, Bruneel SP, Goethals PL, Kopecki I, Elings J, et al. Individual based models for the simulation of fish movement near barriers: Current work and future directions. Journal of environmental management. 2023;335:117538.
7. Alaliyat S, Yndestad H, Davidsen PI. An agent-based approach for predicting patterns of pathogen transmission between aquaculture sites in the Norwegian fjords. Aquaculture. 2019;505:98-111.
8. Halttunen E, Gjelland KO, Glover KA, Johnsen IA, Serra-Llinares RM, Skaala O, et al. Migration of Atlantic salmon post-smolts in a fjord with high infestation pressure of salmon lice. Mar Ecol Prog Ser. 2018;592:243-56. doi: 10.3354/meps12403. PubMed PMID: WOS:000428818700016.
9. Vollset KW. Parasite induced mortality is context dependent in Atlantic salmon: insights from an individual-based model. Sci Rep. 2019;9(1):1-15.
10. Hedger RD, Diserud OH, Finstad B, Jensen AJ, Hendrichsen DK, Ugedal O, et al. Modeling salmon lice effects on sea trout population dynamics using an individual-based approach. Aquaculture Environment Interactions. 2021;13:145-63.
11. Gillibrand PA, Willis KJ. Dispersal of sea louse larvae from salmon farms: modelling the influence of environmental conditions and larval behaviour. Aquatic Biology. 2007;1(1):63-75.
12. Revie CW, Gettinby G, Treasurer JW, Rae GH, Clark N. Temporal, environmental and management factors influencing the epidemiological patterns of sea lice (Lepeophtheirus salmonis) infestations on farmed Atlantic salmon (Salmo salar) in Scotland. Pest Manag Sci. 2002;58(6):576-84. Epub 2002/07/26. doi: 10.1002/ps.476. PubMed PMID: 12138624.
13. Stien A, Bjørn PA, Heuch PA, Elston DA. Population dynamics of salmon lice *Lepeophtheirus salmonis* on Atlantic salmon and sea trout. Mar Ecol Prog Ser. 2005;290:263-75.

14. Kristoffersen AB, Qviller L, Helgesen KO, Vollset KW, Viljugrein HJ, P. A. Quantitative risk assessment of salmon louse-induced mortality of seaward migrating post-smolt Atlantic salmon. Epidemics. 2018;23:19-33.
15. Stige LC, Helgesen KO, Viljugrein H, Qviller L. Modelling salmon lice-induced mortality of wild salmon post-smolts is highly sensitive to calibration data. Aquaculture Environment Interactions. 2022;14:263-77.
16. Stige LC, Helgesen KO, Viljugrein H, Qviller L. A statistical mechanistic approach including temperature and salinity effects to improve salmon lice modelling of infestation pressure. Aquaculture Environment Interactions. 2021;13:339-61.
17. McDaniels T, Wilmot S, Healey M, Hinch S. Vulnerability of Fraser River sockeye salmon to climate change: a life cycle perspective using expert judgments. Journal of Environmental Management. 2010;91(12):2771-80.
18. Johnson B, Gan JC, Godwin SC, Krkosek M, Hunt BP. Juvenile salmon migration observations in the Discovery Islands and Johnstone Strait in British Columbia, Canada in 2018. North Pacific Anadromous Fish Commission. 2019;22.
19. Rechisky EL, Porter AD, Johnston SD, Stevenson CF, Hinch SG, Hunt BP, et al. Exposure time of wild, juvenile sockeye salmon to open- net- pen Atlantic salmon farms in British Columbia, Canada. N Am J Fish Manage. 2021;41(3):650-60.
20. Moriarty M, Ives SC, Murphy JM, Murray AG. Modelling parasite impacts of aquaculture on wild fish: The case of the salmon louse (Lepeophtheirus salmonis) on out-migrating wild Atlantic salmon (Salmo salar) smolt. Preventive Veterinary Medicine. 2023;214:105888.
21. R Core Team. R Development Core Team R: A Language and Environment for Statistical Computing 2023. R Core Team: Vienna, Austria. 2023.
22. Grimm V, Berger U, Bastiansen F, Eliassen S, Ginot V, Giske J, et al. A standard protocol for describing individual-based and agent-based models. Ecol Model. 2006;198(1-2):115-26.
23. Price MH, Proboszcz SL, Routledge RD, Gottesfeld AS, Orr C, Reynolds JD. Sea louse infection of juvenile sockeye salmon in relation to marine salmon farms on Canada's west coast. PLoS One. 2011;6(2):e16851.
24. Hijmans R. raster: Geographic Data Analysis and Modeling_. R package version 3.5-21 2022. Available from: <https://CRAN.R-project.org/package=raster>.
25. Beacham TD, Beamish RJ, Candy JR, Wallace C, Tucker S, Moss JH, et al. Stock-specific migration pathways of juvenile sockeye salmon in British Columbia waters and in the Gulf of Alaska. Trans Am Fish Soc. 2014;143(6):1386-403. doi: 10.1080/00028487.2014.935476.
26. Taylor E, Foote C. Critical swimming velocities of juvenile sockeye salmon and kokanee, the anadromous and non- anadromous forms of Oncorhynchus nerka (Walbaum). J Fish Biol. 1991;38(3):407-19.
27. Johnsen IA, Harvey A, Saevik PN, Sandvik AD, Ugedal O, Adlandsvik B, et al. Salmon lice-induced mortality of Atlantic salmon during post-smolt migration in Norway. ICES J Mar Sci. 2021;78(1):142-54. doi: 10.1093/icesjms/fsaa202. PubMed PMID: WOS:000648942600014.
28. DFO. Industry sea lice counts at BC marine finfish aquaculture sites: Fisheries and Oceans Canada; 2023. Available from: https://open.canada.ca/data/en/dataset/3cafbe89-c98b-4b44-88f1-594e8d28838d.

29. Pécseli H, Trulsen J, Fiksen Ø. Predator–prey encounter and capture rates in turbulent environments. Limnology and Oceanography: Fluids and Environments. 2014;4(1):85-105.
30. Murray AG, Moriarty M. A simple modelling tool for assessing interaction with host and local infestation of sea lice from salmonid farms on wild salmonids based on processes operating at multiple scales in space and time. Ecol Model. 2021;443:109459. doi: 10.1016/j.ecolmodel.2021.109459. PubMed PMID: WOS:000618329100009.
31. Cantrell DL, Rees EE, Vanderstichel R, Grant J, Filgueira R, Revie CW. The sse of kernel fensity rstimation with a bio-physical model provides a method to quantify connectivity among salmon farms: spatial planning and management with epidemiological relevance. Front Vet Sci. 2018;5:269. Epub 2018/11/15. doi: 10.3389/fvets.2018.00269. PubMed PMID: 30425996; PubMed Central PMCID: PMCPMC6218437.
32. Nekouei O, Vanderstichel R, Thakur K, Arriagada G, Patanasatienkul T, Whittaker P, et al. Association between sea lice (*Lepeophtheirus salmonis*) infestation on Atlantic salmon farms and wild Pacific salmon in Muchalat Inlet, Canada. Sci Rep. 2018;8:1-11. doi: ARTN 4023

10.1038/s41598-018-22458-8. PubMed PMID: WOS:000426540800056.
33. van Etten J. R package gdistance: distances and routes on geographical grids. Journal of Statistical Software. 2017;76(13):1-21.
34. Melnychuk MC, Welch DW, Walters CJ. Spatio-temporal migration patterns of Pacific salmon smolts in rivers and coastal marine waters. PLoS One. 2010;5(9):e12916.
35. Samsing F, Solstorm D, Oppedal F, Solstorm F, Dempster T. Gone with the flow: current velocities mediate parasitic infestation of an aquatic host. Int J Parasitol. 2015;45(8):559-65.
36. Buck JC, Lutterschmidt WI. Parasite abundance decreases with host density: evidence of the encounter-dilution effect for a parasite with a complex life cycle. Hydrobiologia. 2017;784:201-10.
37. Bui S, Oppedal F, Nola V, Barrett LT. Where art thou louse? A snapshot of attachment location preferences in salmon lice on Atlantic salmon hosts in sea cages. J Fish Dis. 2020;43(6):697-706.
38. Fast MD. Fish immune responses to parasitic copepod (namely sea lice) infection. Dev Comp Immunol. 2014;43(2):300-12.
39. Anderson JJ, Gurarie E, Zabel RW. Mean free-path length theory of predator–prey interactions: application to juvenile salmon migration. Ecol Model. 2005;186(2):196-211.
40. Barrett LT, Oppedal F, Robinson N, Dempster T. Prevention not cure: a review of methods to avoid sea lice infestations in salmon aquaculture. Reviews in Aquaculture. 2020;12(4):2527-43. doi: https://doi.org/10.1111/raq.12456.
41. Peterman RM, Marinone SG, Thomson KA, Jardine ID, Crittenden RN, Leblond PH, et al. Simulation of juvenile sockeye salmon (*Oncorhynchus nerka*) migrations in the Strait of Georgia, British Columbia. Fish Ocean. 1994;3(4):221-35.
42. Routledge R, Morton A. Effect of government removal of salmon farms on sea lice infection of juvenile wild salmon in the Discovery Islands. Can J Fish Aquat Sci. 2023;(ja).
43. Cantrell D, Vanderstichel R, Filgueira R, Grant J, Revie CW. Validation of a sea lice dispersal model: principles from ecological agent-based models applied to aquatic epidemiology. Aquaculture Environment Interactions. 2021;13:65-79. doi: 10.3354/aei00390. PubMed PMID: WOS:000634851300001.

Figure. 1

Click here to access/download;Figure;Figure 1.tif

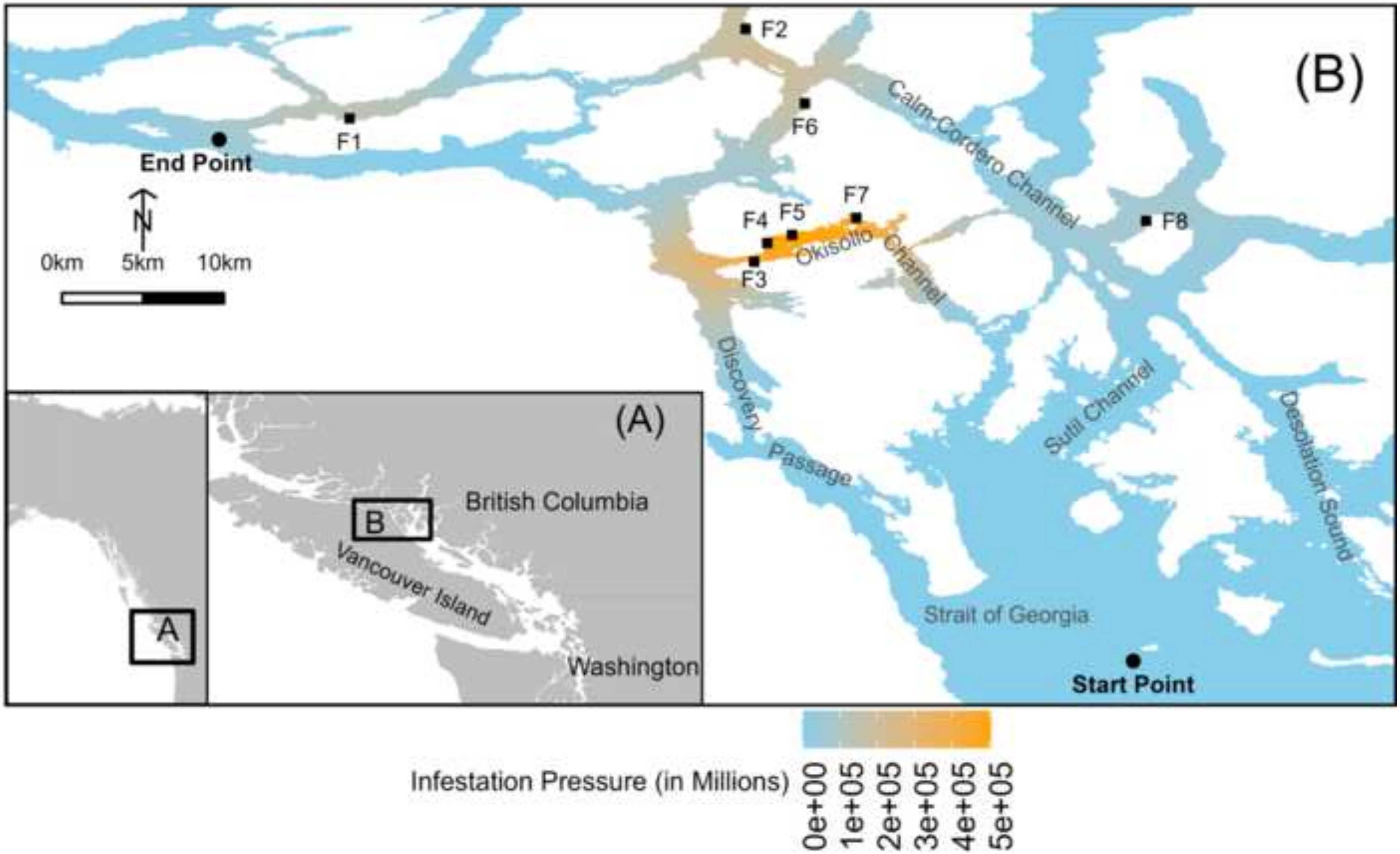

Figure. 2

Click here to access/download;Figure;Figure 2.tif

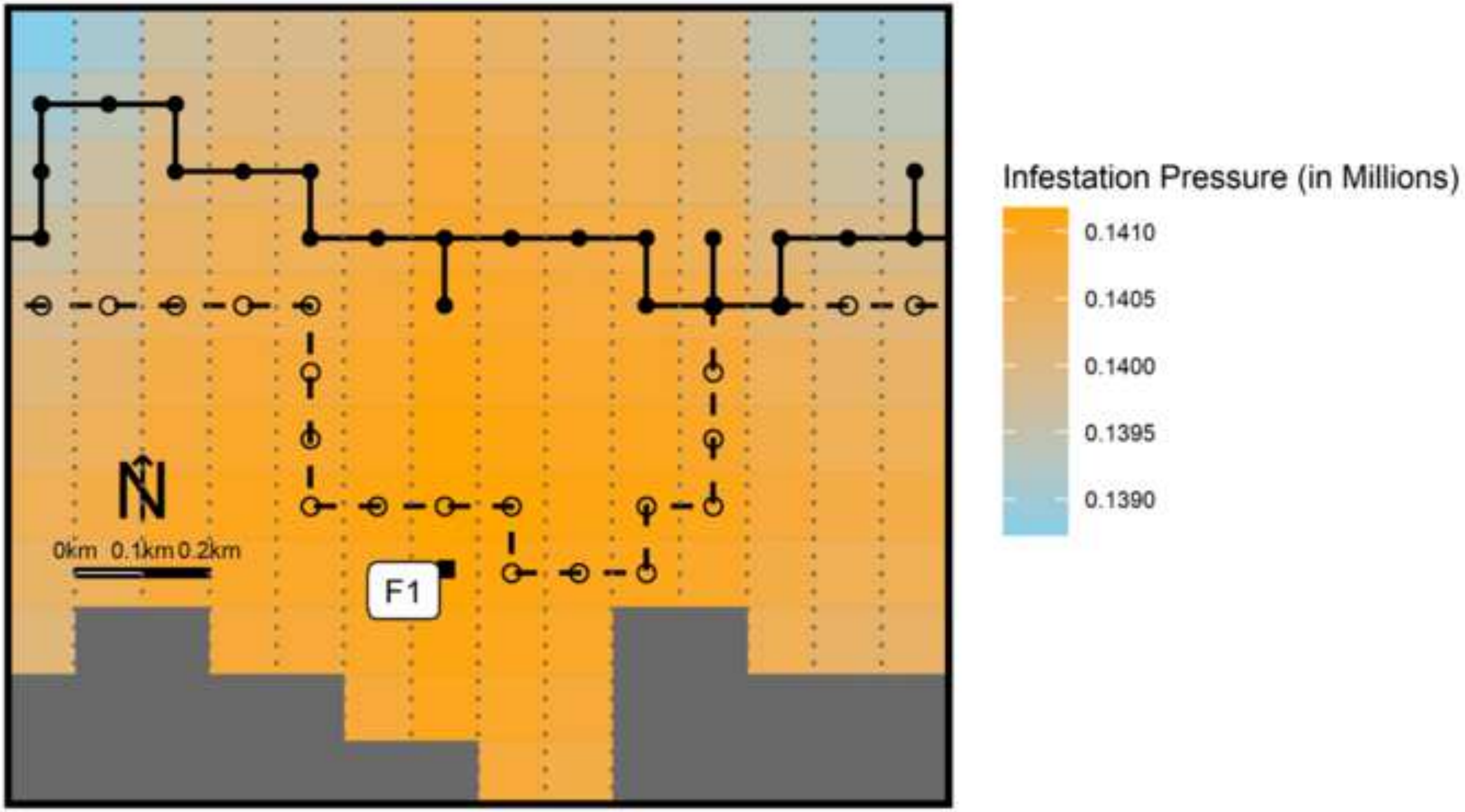

Figure. 3

Click here to access/download;Figure;Figure 3.tif

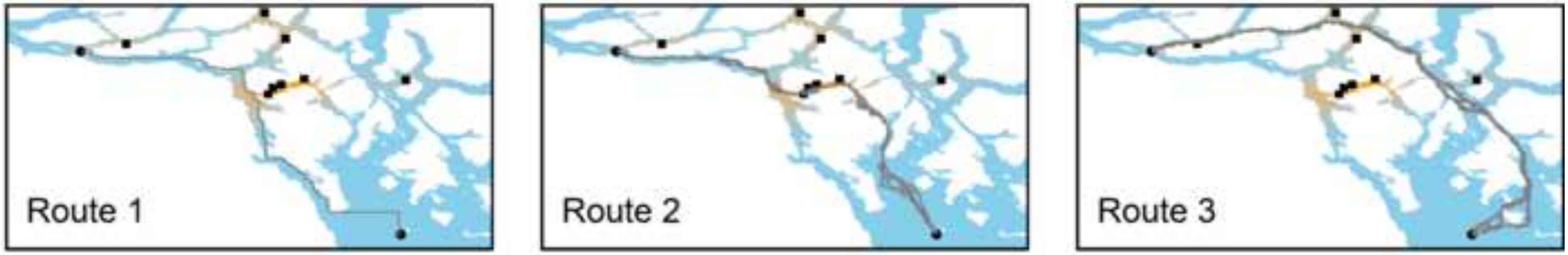

Figure. 4

Click here to access/download;Figure;Figure 4.tif

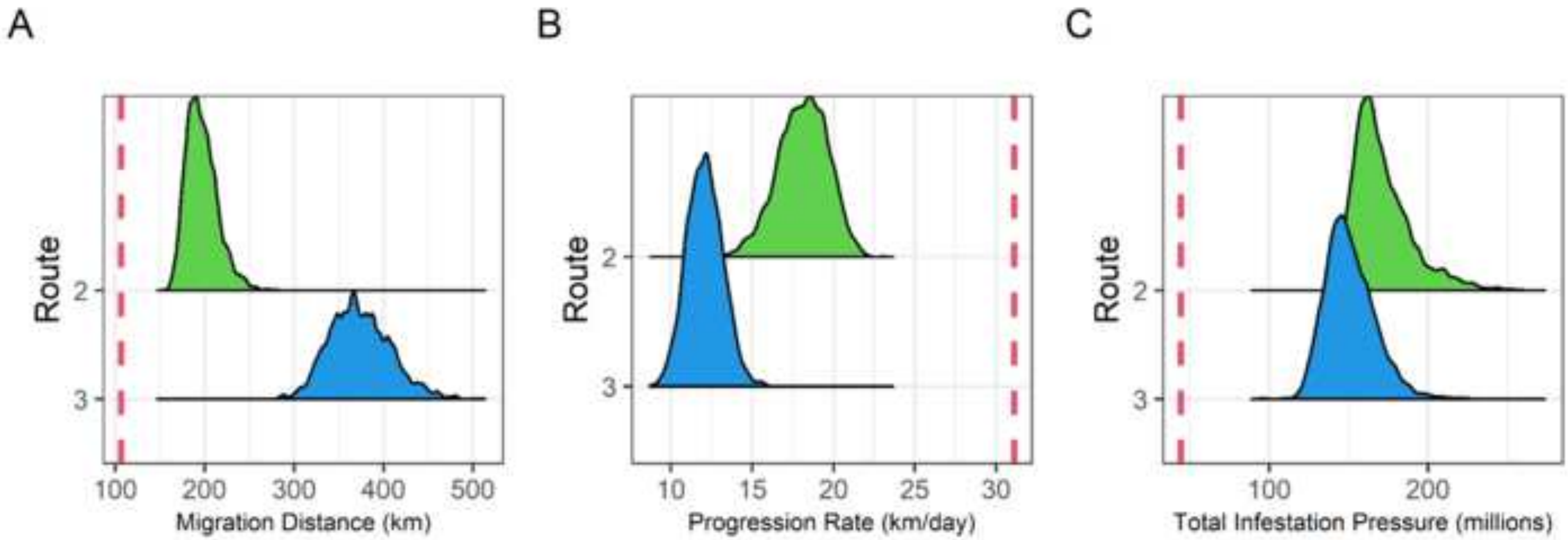

Figure. 5

Click here to access/download;Figure;Figure 5.tif

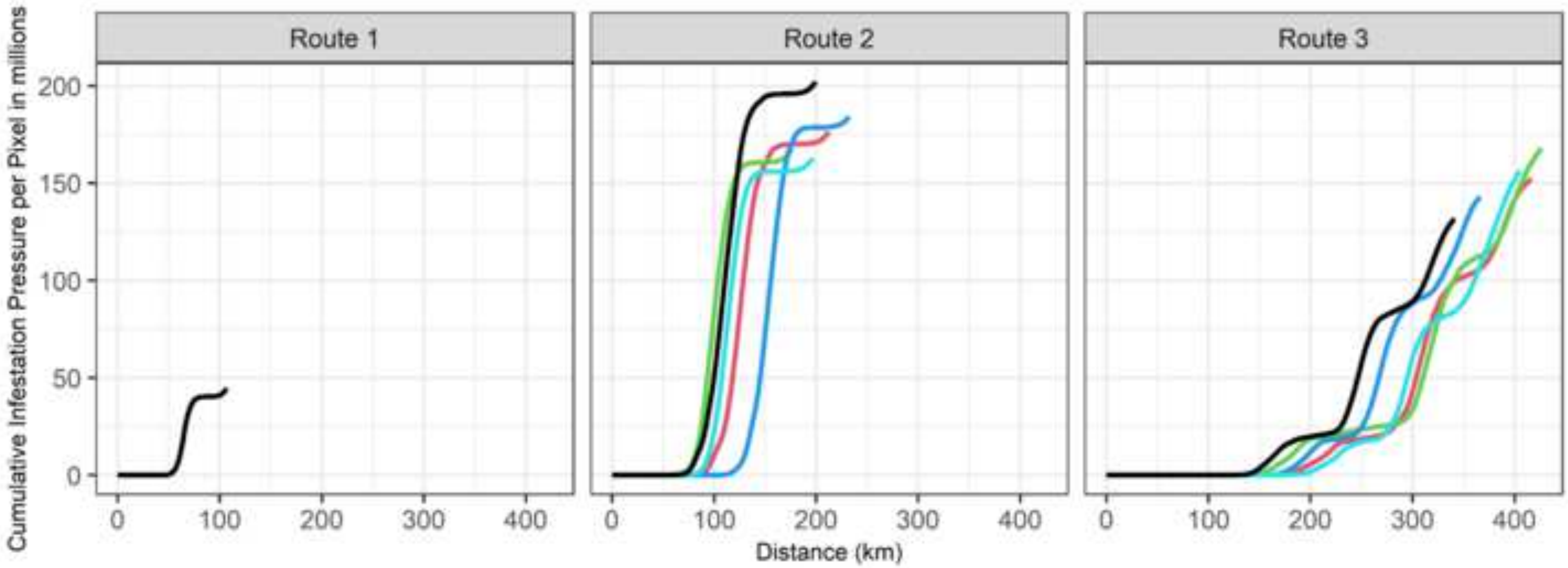

Figure. 6

Click here to access/download;Figure;Figure 6.tif

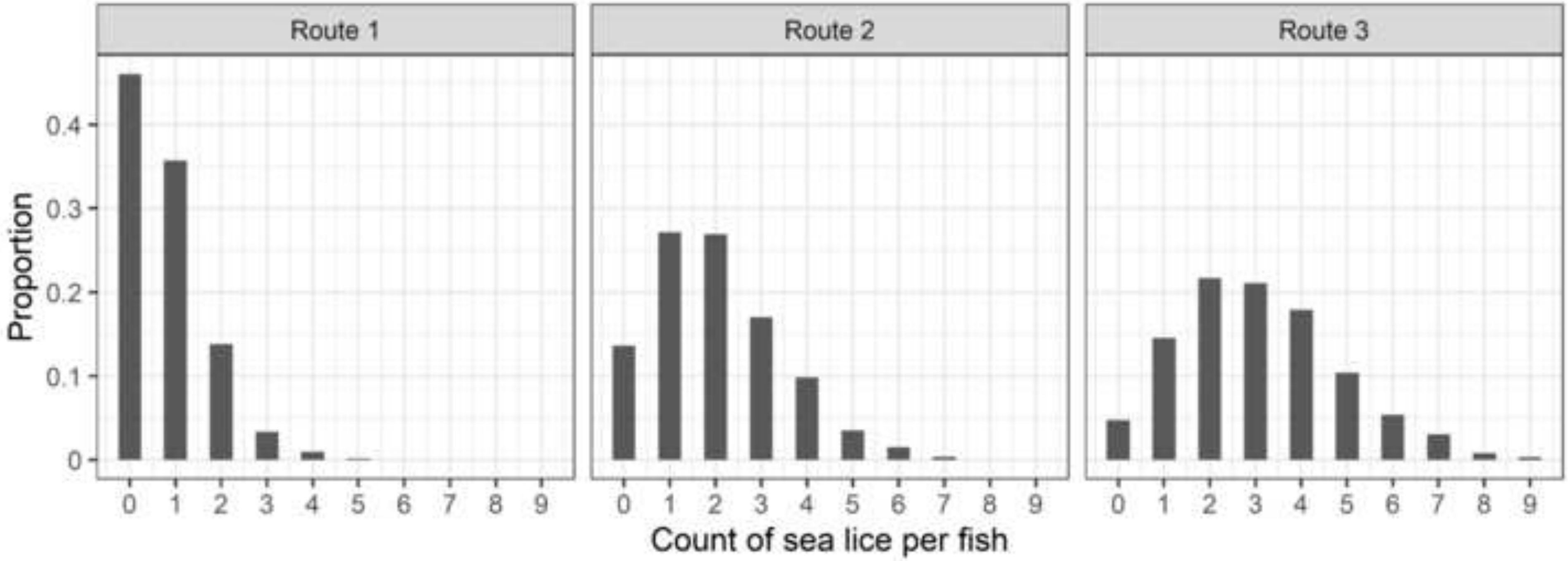

Figure. 7

Click here to access/download;Figure;Figure 7.tif

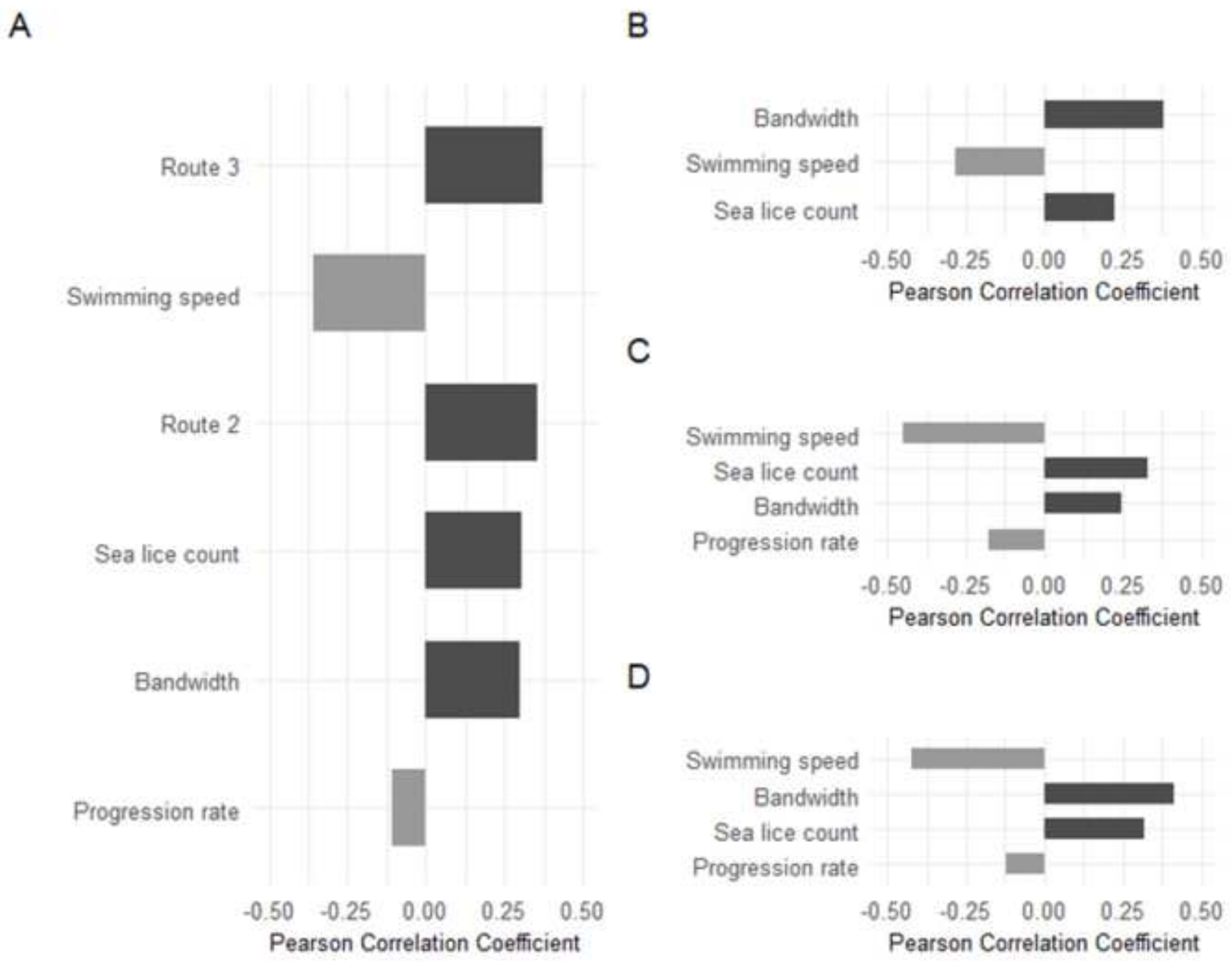

Supplement: Attachment — Submitted filename: PONE-D-24-06375.pdf [file pone.0309215.s002.pdf]
