# Supplementary material for: Wild salmon migration routes influence sea lice infestations: An agent-based model predicting farm-related infestations on juvenile salmon
Source: PLoS One. 2024 Aug 20;19(8):e0309215. doi: 10.1371/journal.pone.0309215 (PMC11335166; doi:10.1371/journal.pone.0309215)
Supplement: Attachment — Submitted filename: Response to Reviewers.docx [file pone.0309215.s003.docx]

May 17, 2024

Journal: PLOS ONE

Manuscript ID: PONE-D-24-06375

Title: “Wild salmon migration routes influence sea lice infestations: An agent-based model predicting farm-related infestations on juvenile salmon”

Thank you for giving us the opportunity to submit a revised draft of our manuscript titled “Wild salmon migration routes influence sea lice infestations: An agent-based model predicting farm-related infestations on juvenile salmon” to PLOS ONE. We appreciate the time and effort that you and the reviewers have dedicated to providing valuable feedback on our manuscript. We are grateful to the reviewers for their insightful comments on our paper. We have now been able to incorporate changes to reflect the suggestions provided by the reviewers, as indicated in the ‘bullet points’ below.

**1. Please ensure that your manuscript meets PLOS ONE's style requirements, including those for file naming. The PLOS ONE style templates can be found at** 
[**https://journals.plos.org/plosone/s/file?id=wjVg/PLOSOne_formatting_sample_main_body.pdf**](https://journals.plos.org/plosone/s/file?id=wjVg/PLOSOne_formatting_sample_main_body.pdf) **and** 
[**https://journals.plos.org/plosone/s/file?id=ba62/PLOSOne_formatting_sample_title_authors_affiliations.pdf**](https://journals.plos.org/plosone/s/file?id=ba62/PLOSOne_formatting_sample_title_authors_affiliations.pdf)

- We ensured that our manuscript meets PLOS ONE's style requirements.

**2. Thank you for stating the following financial disclosure: "Aquaculture, Biotechnology and Aquatic Animal Health Science Branch at the Fisheries and Oceans Canada provided a research supporting fund for this study. "
Please state what role the funders took in the study.  If the funders had no role, please state: ""The funders had no role in study design, data collection and analysis, decision to publish, or preparation of the manuscript."" 
If this statement is not correct you must amend it as needed. 
Please include this amended Role of Funder statement in your cover letter; we will change the online submission form on your behalf.**

- As the funders had no role, we stated: "The funders had no role in study design, data collection and analysis, decision to publish, or preparation of the manuscript." in our cover letter.

**3. We note that Figures 1 and 3 in your submission contain [map/satellite] images which may be copyrighted. All PLOS content is published under the Creative Commons Attribution License (CC BY 4.0), which means that the manuscript, images, and Supporting Information files will be freely available online, and any third party is permitted to access, download, copy, distribute, and use these materials in any way, even commercially, with proper attribution. For these reasons, we cannot publish previously copyrighted maps or satellite images created using proprietary data, such as Google software (Google Maps, Street View, and Earth). For more information, see our copyright guidelines:** [**http://journals.plos.org/plosone/s/licenses-and-copyright**](http://journals.plos.org/plosone/s/licenses-and-copyright)**.**

- The maps in the insets of Figure 1 were created using a shapefile from Natural Earth (public domain): <http://www.naturalearthdata.com/>. This source is recommended by PLOS ONE as an alternative to copyrighted map figures. The maps in Figures 1 and 3 were produced using the SHP file from the British Columbia Data Catalogue: <https://catalogue.data.gov.bc.ca/>, which is also a publicly available domain.
- Therefore, I believe that I do not need to provide a Content Permission Form. If this information is incorrect, please let me know.
- I have included additional descriptions for the map data in the captions of Figures 1 and 3.

**Reviewer #1:**

**Main comments:**

**The sensitivity analysis focuses on the correlation between different model setup parameters and the model output (the number of lice on salmon smolts). That is useful information but somehow difficult to interpret, since the correlation can depend strongly on how large parameter range is explored. Was that range standardised somehow, to make the correlations for different parameters comparable?**

- We agree with your comment. We contemplated how to determine the range of parameters in our sensitivity analysis. Initially, we considered applying an equal ratio to all parameters, for example, from a quarter to four times the baseline values. However, an equal ratio can generate a massively different impact on each parameter. For instance, it is not reasonable to believe that doubling the number of sea lice and doubling the swimming speed would be comparable. An alternative was to use a plausible range for each parameter based on known information, and we chose this alternative. To resolve this issue, we used a probable range for each parameter's values.
- First, the plausible range for the number of sea lice was determined based on the industry sea lice count reported from the farms in the Discovery Islands from 2011 to 2022.
- Second, the plausible range of bandwidth values was determined based on Nekouei et al. (2018).
- Third, the range of swimming speed was determined based on the experiments in Samsing et al. (2015).
- Lastly, the plausible range of progression rates was determined based on our model. Through 3000 simulations, the minimum and maximum progression rates were established as the range of progression rates.
- Specifically, we modified the range of the number of sea lice from one-twelfth to twice the baseline copepodid number to 0.18 to 4.12 times the baseline number. However, the difference in the results was minimal.
- We have rewritten this pertinent section from lines 356 to 365.

**In addition, the correlations do not necessarily show the sensitivity of the conclusions drawn. In particular, I urge to also show results on how conclusions about differences between routes depend on modelling choices.**

- Depending on the chosen model for the relationship between infestation pressure and lice abundance, the results of our modeling varied considerably. Thus, we applied both the 'logarithmic relation' and 'proportional relation' assumptions in our manuscript. Accordingly, we changed Figure 6.
- In Stige et al. (2022), the 'logarithmic model' was proposed as the best-fitting model for their data. Therefore, we placed more emphasis on the 'logarithmic model' than on the 'proportional model'.
- Compared to the logarithmic relationship, the proportional relationship resulted in more occurrences of higher counts of sea lice on fish, and also a higher abundance in Route 2 than in Route 3.
- We have rewritten this pertinent section from lines 311 to 320 and from lines 443 to 455. Additionally, Figure 6 was modified to include the results from the proportional model.

**The sensitivity of the results to the logarithmic relationship between infestation pressure and infestation rate is discussed thoroughly, but as no sensitivity analysis on this model choice is presented, it remains unknown which results are indeed sensitive to this assumption. I strongly suggest to add a sensitivity analysis for this key model assumption. The data for this exist, as reference [31], in addition to the parameters for eq. 3, give parameters for an alternative model formulation that assumes a proportional relationship. The discussion relating to this relationship and the potential influence on results is otherwise very good.**

- We added a sensitivity analysis for the key model assumption, which is the logarithmic relationship between infestation pressure and infestation rate. We used a proportional relationship, which was presented as 'Fixed slope' in Table 2 in Stige et al. (2022). (We assume that Stige et al. (2022), not [31], is the reference you intended to mention.)
- This emphasizes the importance of this assumption in interpreting the outcomes of our model.
- Figure 7 was modified to include the sensitivity analysis using the proportional model.

**Route 1 differs fundamentally from the other two routes in that fish are assumed to take the shortest route without meandering. Is it feasible to add a sensitivity analysis to this assumption, e.g. by assuming a faster swimming speed so that the fish had to meander in all routes? This comment can be ignored if the authors think that such an approach is unlikely to provide new insights.**

- Using the logarithmic model, we considered including meandering in Route 1 as well, because no meandering at all is not realistically true for any route. However, we decided against this because the assumption of no meandering in Route 1 was essential for calculating the observed swimming speed of 31.1 km/day, based on Rechisky et al. (2021).
- We previously explored the relative impacts of various values of swimming speed and progression rate on Routes 2 and 3, as illustrated in the figure below. However, we chose not to include this in our paper because we believe this figure does not provide additional insights beyond those presented in Figure 7. This omitted figure demonstrates that swimming speed is more influential than progression rate, a conclusion also supported by Figure 7. Thus, we did not include this into the manuscript.

Figure 1. Sea lice abundance on juvenile sockeye salmon as they approached the endpoint was analyzed using seven levels of swimming speed and progression rate in the simulations. Note that while the swimming speed values are identical for both routes, the progression rate values differ between the two routes.

**The conclusions about differences in lice infestation along the three routes depend strongly on the progression rates estimated in reference [19]. Please provide a bit more information about sample size and reliability of these estimates in the Material and Methods, and some sentences about this uncertainty in the Discussion.**

- We added the numbers of released and detected fish from lines 262 to 269.
- We discussed the uncertainties in the observations referenced in Rechisky et al. (2021) from lines 604 to 614.

**Line 296: Please explain why you used a dispersion parameter (2.733) from a different model than where the parameters in eq. 3 came from. Consider adding a sensitivity analysis to this modelling choice.**

- We overlooked the need to use the dispersion parameter from equation 3, as specified in the same reference by Stige et al. (2022), in which we newly referred to the dispersion parameter value of 0.921. We have now re-simulated our model with this dispersion parameter and updated all modeling outcomes with these new simulation results.
- The modeling outcome is not sensitive to the values of the dispersion parameter, even if the values are extremely low or high, as illustrated in the figure below. Thus, we did not include this sensitivity analysis in our paper.

Figure 2. Proportions of counts of sea lice per fish as the modelling outcome with varying values of dispersion parameter (rows) in the negative binomial distribution.

**Detailed comments:**

**Line 28: Unclear what “lower-level” means. Is it the same as “low”?**

- “lower-level” adds unnecessary complexity to the sentence. We simplified it by using “low”, as you indicated.

**Line 92: I am not familiar with this species of sea lice. Can the wild salmon also be infested by other louse stages than the larvae? This question is not important for the robustness or validity of the modelling.**

- As the infective stage of Lepeophtheirus salmonis is copepodid, wild salmon are infested with the copepodid stage of Caligus.

**Section 1.2: I missed a description of the time scale of the model (1 out-migration season; time-step 4.63 min.).**

- Fish was assumed to stay at a pixel for 4.63 minutes in a baseline model, which was described in the last paragraph in the section 3.3.1. Also, one out-migration season was assumed.

**Lines 122-125: This list seems a mix of state variables and higher-level model settings, but I am not familiar with how the ODD framework uses the term state variables.**

- From Grimm et al. (2006) “The term ‘state variables’ refers to low-level variables that characterize the low-level entities of the model”.
- We were overly inclusive in our list of state variables for the fish entities as some were constant parameters of the model, and some were calculated values.
- We have corrected the state variable list to: location, days since start, distance travelled, and infestation level.

**Line 130: Each pixel is 100x100 = 10000 m^2, not 100 m^2.**

- It was corrected, as indicated.

**Line 130: Is it correct with plural infestation pressure “values” here? That is confusing.**

- We deleted 'values.' The sentence is clearer without it.

**Line 131: C. clemensi**

- We corrected the part by changing ‘Clemensi’ to ‘clemensi’.

**Line 174: I found this sentence confusing, as I read it as referring to an individual fish, and it was not understandable “consistent” between what. Perhaps rewrite to “... taken by different fish may differ ...” or similar.**

- We corrected the sentence by using ‘differ’.

**Line 197: Is “transmission pressure” the same as other places referred to as “infestation pressure”?**

- You are right. It should be ‘infestation’, not ‘transmission’.

**Line 223: Each pixel is 100x100 = 10000 m^2, not 100 m^2**

- It was corrected as indicated.

**Lines 239-241: This sentence is confusing, as it seems to refer to the migration model in the submitted manuscript. Please make it clear from the beginning of the sentence that this refers to a completely different migration model. It is not at all clear to me why the migration model by Moriarty et al. is discussed here.**

- The sentence was confusing, so we clarified it by emphasizing the balance between the directed path to the predetermined destination and the randomness in their movement. We referred to Moriarty et al. to indicate the integration of these two concepts in fish migrations, in line from 238 to 244.

**Lines 260-261: The rest of the paragraph would be easier to follow if you here wrote more explicitly that acoustically tagged juvenile sockey salmon have been found to have progression rates of X, Y and Z km/day in routes 1, 2, and 3, respectively [19], and that you used these values as basis for the simulations.**

- We added the suggested sentences into the early part of the paragraph.

**Line 286: “after”, or did the salmon reach the lice before they reached the farms?**

- We corrected “before” to “after”.

**Line 297 and elsewhere: I find it confusing that “count” is used as synonym for concentration or number. To me, a count refers to a number obtained by counting something – for example the number of lice counted on a physical sample of fish, which is generally not the case here.**

- We agree with your comment. Thus, throughout the manuscript, we made sure that “count” was used only to indicate the lice attached on fish, and “number” was used to indicate the floating larval lice, which determined the infestation pressure throughout our manuscript.

**Lines 328-354 (Sensitivity analysis): It was hard to understand what was done until after reading the results. It may help if you separate better what was sensitivity to model assumptions (e.g. sea lice abundance in farms), which implied rerunning the model multiple times, and what was postprocessing of model results to look for patterns in the variation between simulated salmon smolts in the baseline run. I am still not sure I have understood exactly how this was done.**

- We re-wrote the paragraph from line 356 to 374, especially focusing the description of how the range of progression rates was assumed through post-processing, compared to the ranges of the other three parameters, which could be pre-determined.

**Lines 339-341: I don’t understand how progression rates and meandering were modelled under the alternative assumptions about swimming speed. Or is this post-processing of results from the baseline model, as described for sensitivity to progression rates?**

- That is post-processing of results from the baseline model. Progression rate cannot be directly assumed, because the rate is the result of simulation. Thus, we used meandering parameter (α) in the simulations to consider the varying values of progression rate in sensitivity analysis. Previous version, the description for this was deficient. We are thankful to you for helping us address this appropriately.
- This has been re-written in line 366 and 374.

**Line 387: How was the total infestation pressure calculated?**

- We added the concise definition of the total infestation pressure in the sentence.

**Lines 489-500: This paragraph does not seem to distinguish well between the total infestation, which is addressed by the sensitivity analysis, and infestation rate, which is addressed in the experiments in reference [35]. Please rewrite for clarity.**

- We rewrote this paragraph to improve clarity. We used ‘the number of sea lice on fish’ to indicate our modelling outcome, while we mentioned ‘infestation rates’ to indicate the outcome in the experiment in Samsing *et al*.

**Also, please write out the author (Samsing et al.) in the first sentence around line 489, so that it is immediately clear that the results discussed around line 499 is the same study and not another.**

- We mentioned the author (Samsing et al.) in the earlier part in the paragraph.

**Lines 501-502 seems to repeat previous points and lines 502-505 seem to be the mechanism behind [35]’s findings about infestation rate, and not “In addition”.**

- We rewrote this paragraph, especially removing redundant parts.

**Lines 523-525: It is a misunderstanding that reference [15] required choosing between calibrating to trawl data and 10 km/day OR sentinel cage data and 7 km/day. Both trawl and cage data were used for both speeds, and the choice of which data to calibrate to can be made by setting the indicator variable for cage vs trawl to 0 or 1.**

- We revised the paragraph by removing the second reason, which incorrectly referred [15].

**Reviewer #2:**

**1. Re-phrase the sentences**

**line 26-28 "Specifically, salmon traveling longer migration routes farther from farms may experience higher sea lice loads than those on shorter, farm-adjacent routes"**

**This sentence may mislead the readers given that the shorter route is already allocated the higher progression rate with zero alpha based on previous studies. The results may only discuss about the progression rate and infestation levels rather than their proximity to farms.**

- We agree with this. The sentence may cause confusion to readers by mentioning even the proximity to farms, which was not investigated in our study. Thus, we rephrased the sentence by replacing proximity with infestation pressure.

**2. Abbreviations**

**line 58: ABMs -**

**Avoid plurals in abbreviations**

- We corrected this part, as indicated.

**3. Consistency**

**line 139 and 146: Fraser River and Fraser river**

**Adhere to one form when indicating the names of the places**

- We changed ‘Fraser river’ to ‘Fraser River’.

**4. Syntax**

**line 145: 1st – superscript**

- In lines 124 and 145, they were corrected as “June 1^St^ ”.

**line 198: "Details" - Please use the section number along with the section heading**

- We changed the part as “in Section 3 ‘Details’, including the section number.

**line 339; "British Columbia"**

**Already defined as BC and yet it has been expanded again**

- “British Columbia” in line 285 and 339 was changed to BC.

**line 552: "sockeye"**

**Capitalise the first letter of a sentence**

- We corrected the part, as indicated.
