# Supplementary material for: Wild salmon migration routes influence sea lice infestations: An agent-based model predicting farm-related infestations on juvenile salmon
Source: PLoS One. 2024 Aug 20;19(8):e0309215. doi: 10.1371/journal.pone.0309215 (PMC11335166; doi:10.1371/journal.pone.0309215)
Supplement: Attachment — Submitted filename: Response to Reviewers.docx [file pone.0309215.s004.docx]

July 22, 2024

Journal: PLOS ONE

Manuscript ID: PONE-D-24-06375R1

Title: “Wild salmon migration routes influence sea lice infestations: An agent-based model predicting farm-related infestations on juvenile salmon”

Thank you for giving us the opportunity to re-submit a revised draft of our manuscript titled “Wild salmon migration routes influence sea lice infestations: An agent-based model predicting farm-related infestations on juvenile salmon” to PLOS ONE. We appreciate the time and effort that you and the reviewers have dedicated to providing valuable feedback on our manuscript. We are grateful to the reviewers for their insightful comments on our paper.

**1) I think the limitations of the infestation model should be clarified better:**

**- Around L 315 (“lice data”), it should be clarified that the infestation model is based on data on salmon lice Lepeophtheirus salmonis infestations on juvenile Atlantic salmon Salmo salar.**

- We made clear the equation is based on Lepeophtheirus salmonis infestations on juvenile Atlantic salmon Salmo salar.

**- Around L 625-630, where model limitations are discussed, I think it should be mentioned that to accurate assess the risk in a specific area, it would be necessary with detailed data on spatiotemporal variation in infestation pressure and an infestation model calibrated to observations of Caligus clemensi sea lice on juvenile sockeye salmon Oncorhyncus nerka.**

- We agree and updated the part to emphasize the need for detailed spatiotemporal data and a model calibrated to observations of specific sea lice and salmon species to accurately assess risks in specific areas.

**This limitation is not relevant for the conclusions drawn in this paper, but relevant to mention since the model presents the model for the first time, and the purpose of the model as given on L 110-111 seems to open for applying the model for a somewhat wider purpose.**

- With your comment, we clarified the applicability of our model for broader purposes in the part of Purpose.

**2) I find the text now generally very clear. The explanation on L 376-383 can however be made clearer by explaining that the random draw of values for the four parameters was done jointly, resulting in 3000 simulations where all four parameters varied randomly within the specified ranges.**

- We revised the paragraph to clarify that 3000 simulations were conducted, each with random values for the four parameters.

**3) Figure 7 legend needs to explain the difference between A-1 and A-2 etc.**

- We updated the legend for Figure 7 to clarify the distinctions between labels 1 and 2. Additionally, we revised the corresponding sections of the text.

**4) L 584-588: An implicit assumption of this argumentation is that lice interfere with one another if their zones overlap, so that a louse has reduced chance of infesting a fish if it is close to another louse. Otherwise, intersecting zones do not reduce the expected number of infestations compared to non-intersecting zones (but the variance is increased). This assumption should be made explicit and the underlying mechanism explained, and if possible supported by references.**

- We revised the paragraph, explicitly mentioning the suggested assumption.

**5) I noted a few possible minor errors:**

**L 181: this --> these**

- We made the corrections as indicated.

**L 252: “k and l” --> “l and m”, for consistency with eq. 2?**

- We made the corrections as indicated.

**L 293 – 295: “they” on L 295 does not point back on anything. Rewrite first sentence to start, e.g., with** **“Price et al demonstrated markedly...”?**

- We made clear who ‘they’ is indicating.

**L 322: Quotation marks around ‘proportional model’ are missing**

- We made the corrections as indicated.

**L 411: This --> These**

- We made the corrections as indicated.

**L 428: the smaller --> smaller**

- We made the corrections as indicated.
